# The Role of a C‐Terminal Seven‐Amino Acid Motif in TbCSV C3 Protein and Its Interaction With NbPOLA2 in Enhancing Viral Replication

**DOI:** 10.1111/mpp.70068

**Published:** 2025-03-02

**Authors:** Mingjun Li, Puxin Huang, Zhou Jia, Xinyuan Lang, Lyuxin Wang, Miao Sun, Hussein Ghanem, Gentu Wu, Ling Qing

**Affiliations:** ^1^ Chongqing Key Laboratory of Plant Disease Biology, College of Plant Protection Southwest University Chongqing China; ^2^ Key Laboratory of Agricultural Biosafety and Green Production of Upper Yangtze River (Ministry of Education) Southwest University Chongqing China; ^3^ College of Biology and Food Engineering Chongqing Three Gorges University Chongqing China; ^4^ National Citrus Engineering Research Center Southwest University Chongqing China

**Keywords:** amino acid motif, C3 protein, DNA polymerase, geminivirus, replication enhancement, tobacco curly shoot virus, viral replication

## Abstract

The C3 protein of tobacco curly shoot virus (TbCSV), a possible evolutionary intermediate between truly monopartite begomoviruses and those requiring satellite molecules for infectivity, has been identified as a viral replication enhancer (REn). However, the mechanisms underlying this enhancement are largely unknown. In this study, we generated two mutant infectious clones of TbCSV: one with a deletion of the 3′ end region of the *C3* gene that does not overlap with *C2* (TbCSV_dC3_) and another in which this region was replaced by a phylogenetically unrelated *iLOV* gene sequence (TbCSV_dC3‐iLOV_). Our findings highlight the crucial role of the 3′ end region of *C3* for viral DNA accumulation and further demonstrated that overexpression of TbCSV C3 protein in trans complements the functional deficiency of TbCSV_dC3_. Further analyses revealed the essential role of the C‐terminal seven‐amino acid motif from residues 123–129 of C3 in replication enhancement. Previous studies suggested that both intra‐ and intermolecular interactions of C3/AC3 proteins encoded by some other geminiviruses are vital for their capacity to enhance replication. Interestingly, among the tested potential interactors, NbPOLA2, a subunit of DNA polymerase α, was confirmed to interact with C3 in yeast and in planta. Our findings indicated that NbPOLA2 positively regulates TbCSV replication and infection and that the seven‐amino acid motif (residues 123–129) in C3 is required for recruiting NbPOLA2 to facilitate TbCSV replication by mediating the viral double‐stranded DNA (dsDNA) replication intermediate synthesis. These findings contribute to our understanding of the mechanisms through which the C3 protein enhances TbCSV replication.

## Graphical abstract

A C‐terminal seven‐amino acid motif in C3 protein of tobacco curly shoot virus is essential for viral replication enhancement, recruiting NbPOLA2 to facilitate the viral double‐stranded DNA replication intermediate synthesis.

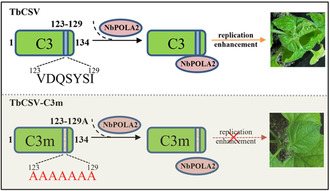

## 1 Introduction

Geminiviruses are a large family of plant viruses that cause severe diseases in numerous crops worldwide, particularly in tropical and subtropical regions, resulting in substantial agricultural losses (Zhou 2013; Li, Qiao, et al. 2022). Members of the *Geminiviridae* family possess a circular single‐stranded DNA (ssDNA) genome that is encapsidated within twinned icosahedral particles (Hanley‐Bowdoin et al. 2013). Currently, this family is classified into 14 genera: *Becurtovirus*, *Begomovirus*, *Capulavirus*, *Citlodavirus*, *Curtovirus*, *Eragrovirus*, *Grablovirus*, *Maldovirus*, *Mastrevirus*, *Mulcrilevirus*, *Opunvirus*, *Topilevirus*, *Topocuvirus* and *Turncurtovirus*, with *Begomovirus* being the largest genus comprising 445 members (https://ictv.global/taxonomy). Begomoviruses are further classified into two groups: bipartite viruses, which possess two genomic components (DNA‐A and DNA‐B), and monopartite viruses, which contain a single genomic molecule (Rojas et al. 2005). With some monopartite begomoviruses, a circular ssDNA satellite molecule is also required for successful infection (Yang et al. 2019; Zhou 2013). The DNA‐A component of bipartite begomoviruses in the ‘Old World’ and the genome of monopartite begomoviruses were originally believed to encode six proteins (V1/AV1 and V2/AV2 from the viral strand, and C1/AC1, C2/AC2, C3/AC3 and C4/AC4 from the complementary strand) (Fondong 2013). Recently, several additional proteins have been identified, including V3, C5/AC5, C6 and C7 (Gong et al. 2021; Gong et al. 2022; Li et al. 2015; Li, Su, et al. 2021; Liu et al. 2023; Wang, Wang, et al. 2022). The DNA‐B molecule of bipartite begomoviruses encodes two proteins, BV1 and BC1 (Fondong 2013). These viral proteins function in diverse cycle processes, such as viral replication, transcription, encapsidation, movement and pathogenicity (Fondong 2013; Gong et al. 2022; Li, Su, et al. 2021).

Geminiviral replication occurs in the nuclei of infected plant cells, through rolling circle replication (RCR) or recombination‐dependent replication (RDR), using the host replication machinery (Hanley‐Bowdoin et al. 2000; Jeske et al. 2001). Notably, among all the viral proteins, only the C1/AC1 (replication associated protein, Rep) is reported to be indispensable for geminiviral RCR, which functions in the initiation and elongation of replication and the release of newly synthesised viral genomes (Rizvi et al. 2015; Hanley‐Bowdoin et al. 2013). Another viral protein, C3/AC3, acts as a replication enhancer (REn), and the null mutation of this protein results in decreased geminiviral genome accumulation (Settlage et al. 2005; Sun et al. 2020; Pasumarthy et al. 2011; Morris et al. 1991). Previous reports have shown that the oligomerisation of C3/AC3 and the hetero‐oligomerisation of C3/AC3 with C1/AC1 are essential for the replication enhancement function in several begomoviruses, including tomato yellow leaf curl virus (TYLCV), tomato golden mosaic virus (TGMV) and bean golden mosaic virus (BGMV) (Settlage et al. 2005; Settlage et al. 1996). In addition, the interactions between C3/AC3 and a few host factors, such as proliferating cell nuclear antigen (PCNA), retinoblastoma‐related protein (pRBR) and NAC domain‐containing transcription factor, have been reported to contribute to its replication enhancement activity (Selth et al. 2005; Castillo et al. 2003; Settlage et al. 2001). Moreover, recent findings by Wu et al. (2021) revealed that the subunits of host nuclear replicative DNA polymerases α (POLA2) and δ (POLD2) can be directly recruited by the C3 protein of TYLCV and function in viral replication. Previous studies on TYLCV and TGMV identified four critical domains in C3/AC3 proteins responsible for protein interactions with viral and host factors and proposed a C3 interaction model (Settlage et al. 2005). However, given the high variation in the amino acid sequences of geminiviral C3/AC3 proteins, the crucial motifs in the C3 protein that have biological significance in its replication enhancement activity are worth investigating for different viruses.

Tobacco curly shoot virus (TbCSV), a member of the genus *Begomovirus* in the family *Geminiviridae*, is prevalent in southwest China, causing leaf curl disease on tobacco and tomato crops (Li et al. 2002; Xie et al. 2002). While a small proportion of TbCSV isolates are associated with a β‐satellite DNA molecule in the field, the DNAβ is dispensable for TbCSV infection (Li et al. 2005). As such, TbCSV is considered an evolutionary intermediate between the truly monopartite begomoviruses and those requiring a DNAβ for successful infection. Our previous research demonstrated that a mutation in the start codon of the TbCSV C3 open reading frame (ORF) reduced viral DNA accumulation, whereas it only slightly delayed the onset of viral symptoms (Sun et al. 2020). In this study, we illustrate that deletion or replacement of the 3′ end region of the TbCSV *C3* gene, which does not overlap with the *C2* gene sequence, significantly reduces viral accumulation and viral disease severity. Using a mutant infectious clone with obviously decreased infectivity, we further determined the nucleotide region 367–387 of the *C3* gene, corresponding to amino acid residues 123–129, is indispensable for the proviral function of *C3* in enhancing viral replication. Protein interaction analysis revealed that the seven‐amino acid motif spanning residues 123–129 of TbCSV C3 is pivotal for mediating the interaction between C3 and NbPOLA2, a subunit of DNA polymerase α that facilitates TbCSV replication and infection.

## 2 Results

### 2.1 The 3' End Region of TbCSV *C3* Gene Is Essential for Robust Viral Accumulation and Infection

**FIGURE 1:**
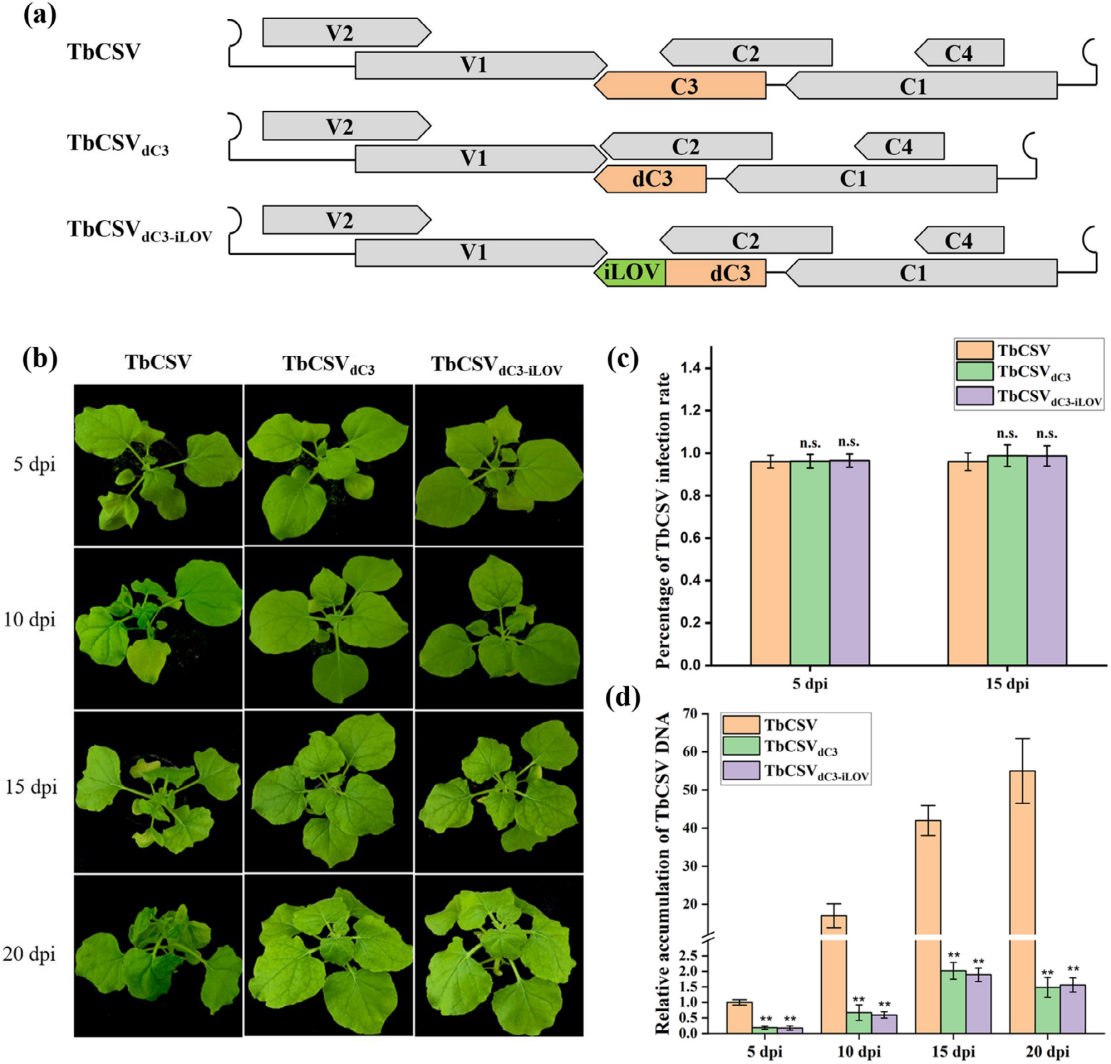
Deletion or replacement of the 3′ end region of the TbCSV *C3* gene attenuates viral infection. (a) Schematic diagram of the TbCSV, TbCSV_dC3_ and TbCSV_dC3‐iLOV_ genomes. (b) Symptoms of *Nicotiana benthamiana* plants inoculated with TbCSV, TbCSV_dC3_ or TbCSV_dC3‐iLOV_ at 5, 10, 15 and 20 days post‐inoculation (dpi). The results were reproduced in three independent experiments using 12 plants per treatment. (c) Infection rate of TbCSV, TbCSV_dC3_ and TbCSV_dC3‐iLOV_ in inoculated *N. benthamiana* plants, determined by PCR detection of the virus in upper new leaves at 5 and 15 dpi. Data are presented as the means from three independent experiments. Bars labelled with ‘n.s.’ indicate no significant difference (*p* > 0.05, *t* test). (d) Relative accumulation of TbCSV DNA in TbCSV‐, TbCSV_dC3_‐ and TbCSV_dC3‐iLOV_‐inoculated *N. benthamiana* plants at 5, 10, 15 and 20 dpi. Four plants were analysed for each treatment, and three technical replicates were used for each biological sample. Error bars represent means ± SEM. Significant differences were determined using Student's *t* test, **p < 0.01. *Nicotiana benthamiana* 25S ribosomal RNA was used as the internal reference. The experiment was repeated three times with similar results.

In our previous study, we demonstrated that a C3 null mutant virus, TbCSV_∆C3_, lacking two potential start codons, led to a significant reduction in TbCSV DNA accumulation. However, it only caused a slight delay and attenuation in symptom expression in infected *Nicotiana benthamiana* plants (Sun et al. 2020). Earlier reports showed that deletions of the 3′ end sequence in the *AL3* gene of TGMV hampered viral DNA accumulation and notably altered the timing and severity of symptoms (Elmer et al. 1988; Settlage et al. 2005). To investigate the functional significance of the 3′ end sequence of the TbCSV *C3* gene, we constructed two mutant infectious clones: TbCSV_dC3_, lacking the 3′ end 144 nucleotides (from nucleotide 262 to 405) that do not overlap with the C2‐encoding region, and TbCSV_dC3‐iLOV_, where the 3′ end sequence was replaced by an exogenous *iLOV* sequence (Figure 1a). Subsequently, TbCSV_dC3_ and TbCSV_dC3‐iLOV_ mutants were agroinoculated into *N. benthamiana* plants, with the inoculation of wild‐type TbCSV serving as a control. As reported previously, *N. benthamiana* plants infected with wild‐type TbCSV exhibited a leaf curling phenotype in upper leaves as early as 5 days post‐inoculation (dpi), with symptoms intensifying over time (Figure 1b). In contrast, plants infected with TbCSV_dC3_ and TbCSV_dC3‐iLOV_ mutants exhibited only mild curling of the uppermost leaves, with no obvious abnormal developmental phenotype, even at 20 dpi (Figure 1b).

To assess systemic infection, we collected the upper new leaves from plants inoculated with TbCSV, TbCSV_dC3_ or TbCSV_dC3‐iLOV_ at 5 and 15 dpi and subjected to PCR detection of virus infections. Our results demonstrated that all three viruses could systemically infect *N. benthamiana* plants (Figure 1c). However, quantitative PCR (qPCR) analysis revealed significantly lower viral DNA accumulation in plants inoculated with TbCSV_dC3_ and TbCSV_dC3‐iLOV_ compared to wild‐type TbCSV (Figure 1d). Taken together, these findings demonstrate that deletion or replacement of the 3′ end 144 nucleotides of the *C3* gene (from nucleotide 262 to the end) impairs viral accumulation and infection.

### 2.2 Trans Expression of C3 Rescues the Infection Ability of TbCSV_dC3_ Mutant

**FIGURE 2:**
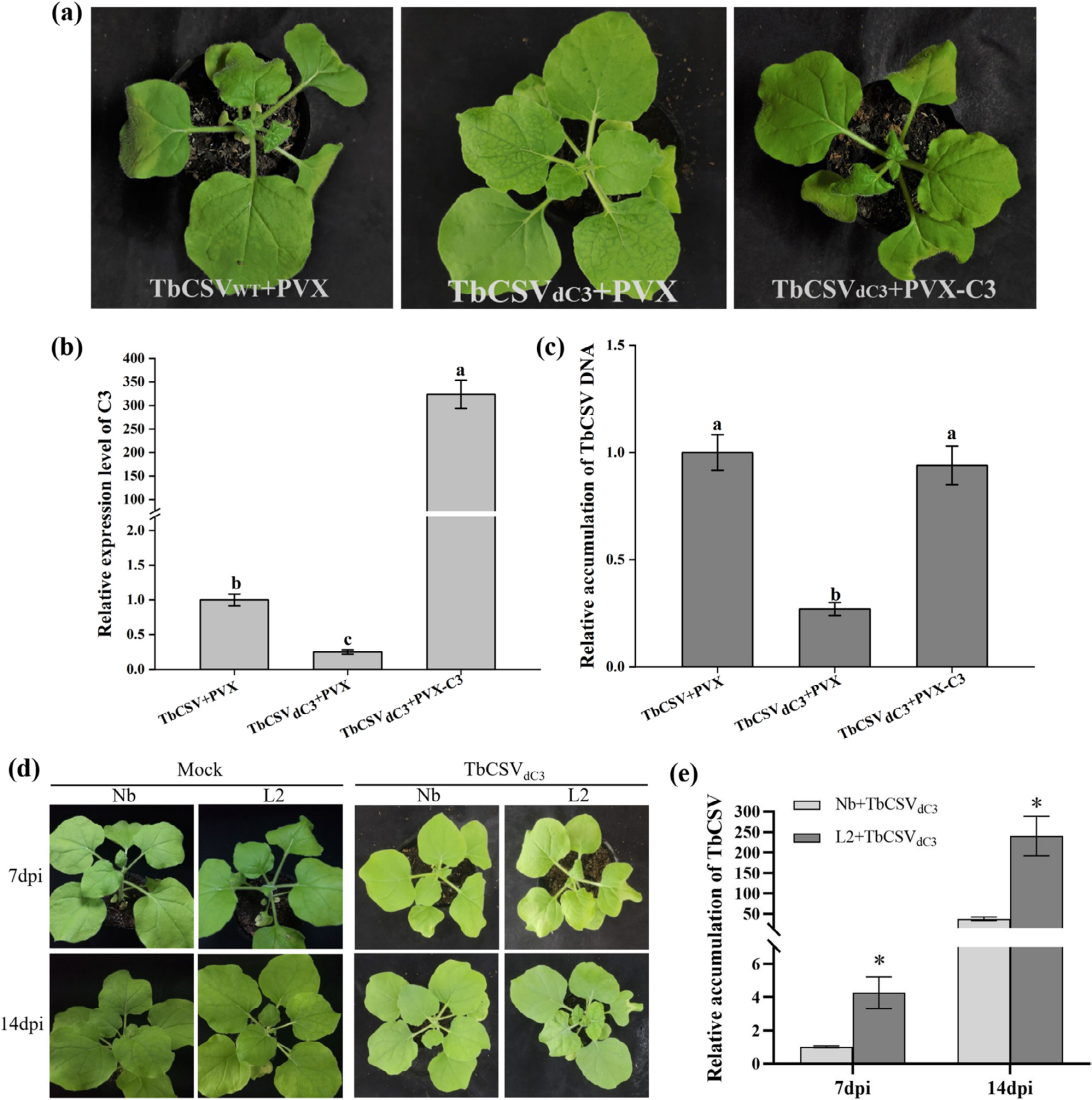
Trans expression of C3 rescues the functional deficiency of TbCSV_dC3_ mutant. (a–c) TbCXV *C3* systemically overexpressed by virus vector PVX facilitates TbCSV_dC3_ mutant infection. (a) Symptoms of *Nicotiana benthamiana* plants co‐infected with TbCSV/PVX, TbCSV_dC3_/PVX or TbCSV_dC3_/PVX‐C3 at 7 days post‐inoculation (dpi). (b) Reverse transcription‐quantitative PCR (RT‐qPCR) analysis of the relative expression levels of *C3*. *NbActin* was used as the internal reference. (c) Relative accumulations of TbCSV DNA. (d, e) Complementation of TbCSV_dC3_ in *C3*‐overexpressing transgenic *N. benthamiana* plants. (d) Symptoms induced by TbCSV_dC3_ infection on wild‐type (Nb) and *C3*‐overexpressing transgenic *N. benthamiana* plants (L2) at 7 and 14 dpi. (e) Relative accumulations of TbCSV DNA. In (c) and (e), *N. benthamiana* 25S ribosomal RNA was used as the internal reference. Total RNA and DNA were extracted from the upper systemically infected leaves and subjected to RT‐qPCR (b) and qPCR (c, e). In (a) and (d), the results were reproduced in three independent experiments using 12 plants per treatment. In quantitative experiments, four plants were analysed for each treatment and three technical replicates were used for each biological sample. Error bars represent means ± SEM. The different letters above each bar in panels (b) and (c) indicates statistically significant differences as determined by one‐way analysis of variance followed by Tukey's multiple test (*p* < 0.05). The ‘*’ above the bars in panel (e) indicates statistically significant differences as determined by Student's *t* test (*p* < 0.05).

In view of the similar functional deficiency of TbCSV_dC3_ and TbCSV_dC3‐iLOV_ mutants, TbCSV_dC3_ was selected for further investigations. The coding sequence of TbCSV C3 was inserted into the potato virus X (PVX) vector (PVX‐C3) and co‐inoculated with TbCSV_dC3_ into *N. benthamiana* plants. Co‐infection with wild‐type PVX and TbCSV or TbCSV_dC3_ served as control. At 7 dpi, downward curling of the upper new leaves could be readily observed in TbCSV_dC3_/PVX‐C3 co‐infected plants, as that shown in TbCSV/PVX‐inoculated control plants. In contrast, except for the mosaic phenotype induced by PVX infection, there was no visible leaf curling symptom on TbCSV_dC3_/PVX‐infected plants (Figure 2a). qPCR analyses showed that the viral DNA in systemically infected leaves of TbCSV_dC3_/PVX‐C3 co‐inoculated plants accumulated a similar level to that in TbCSV/PVX control, and accumulated approximately threefold higher than that in TbCSV_dC3_/PVX co‐infected control plants (Figure 2c). Furthermore, the expression levels of the *C3* gene were determined by reverse transcription (RT)‐qPCR, and our results showed more than 300‐fold increase in *C3* transcript levels in PVX‐C3‐infected plants over that in PVX‐infected controls (Figure 2b).

We have known that the viral accumulation of PVX can also be elevated when it was used to mediate TbCSV *C3* overexpression (Sun et al. 2020). To exclude the potential effects of differentially accumulated PVX on TbCSV_dC3_ infection, transgenic *N. benthamiana* plants stably expressing the TbCSV *C3* gene were generated. The T_2_ generation plants derived from the *C3*‐overexpressing line L2 were inoculated with TbCSV_dC3_, and the wild‐type *N. benthamiana* plants served as control. Consistent with the PVX‐mediated trans‐expression assay, more severe viral symptoms manifested on *C3*‐expressing transgenic plants at 7 and 14 dpi (Figure 2d). Correspondingly, viral DNA accumulation levels increased approximately four‐ and eightfold in transgenic plants compared to those in wild‐type *N. benthamiana* plants at 7 and 14 dpi, respectively (Figure 2e). High levels of *C3* expression in transgenic plants were confirmed by RT‐qPCR (Figure S1). These findings demonstrate that the trans‐expression of C3 protein is able to complement the functional deficiency of the TbCSV_dC3_ mutant, which lacks the 3′ end region of the *C3* gene and exhibits reduced virus accumulation in *N. benthamiana* plants.

### 2.3 The C‐Terminal Amino Acid Motif From Residues 123 to 129 of TbCSV C3 Is Critical for Its Replication Enhancement Activity

**FIGURE 3:**
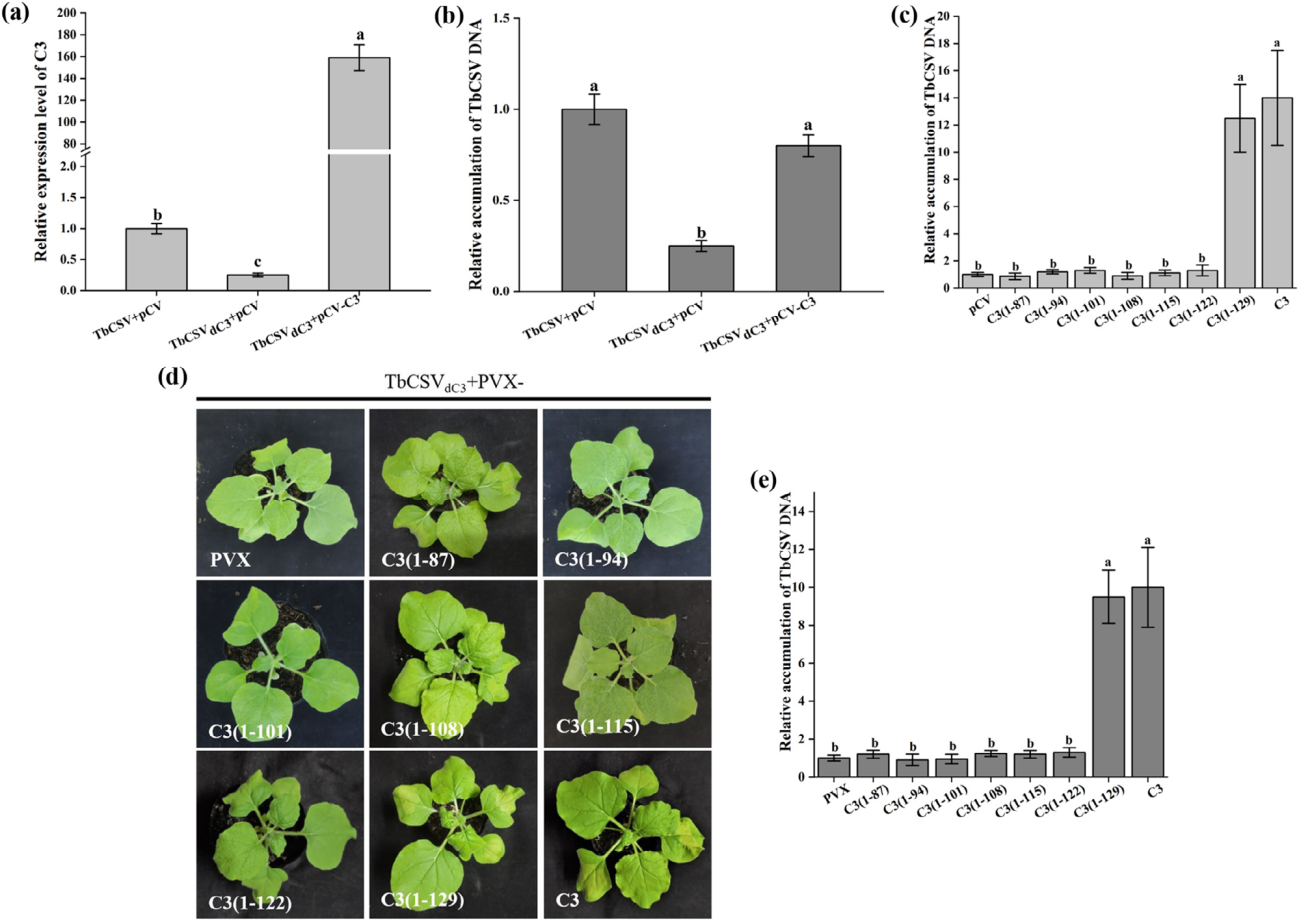
The C‐terminal 123–129 amino acids of TbCSV C3 protein are critical for its replication enhancement ability. (a, b) Transient expression of *C3* elevated the replication level of TbCSV_dC3_. (a) Reverse transcription quantitative PCR analysis of the relative expression levels of *C3* using *NbActin* as the internal reference. (b) Relative accumulations of TbCSV DNA. (c) Relative accumulations of TbCSV DNA in *Nicotiana benthamiana* leaves transiently expressing *C3* or truncated *C3* mutants. (d) Symptoms of *N. benthamiana* plants co‐infected with TbCSV_dC3_ and PVX‐C3 or truncated C3 mutants. The results were reproduced in three independent experiments using 12 plants per treatment. (e) Relative accumulations of TbCSV DNA in infected *N. benthamiana* plants. *N. benthamiana* 25S ribosomal RNA was used as the internal reference in (b), (c) and (e). In quantitative experiments, four plants were analysed for each treatment, and three technical replicates were used for each biological sample. Error bars represent means ± SEM. The different letters above each bar in panels (a), (b), (c) and (e) indicate statistically significant differences as determined by one‐way analysis of variance followed by Tukey's multiple test (*p* < 0.05).

Previous reports revealed that the C3 protein enhances TbCSV replication, prompting us to investigate whether the decreased accumulation of TbCSV_dC3_ can be attributed to the impaired virus replication, using a local infection assay as described previously with minor modifications (Sun et al. 2020; Wu et al. 2019; Wu et al. 2021; Chang et al. 2022). Briefly, the fully expanded *N. benthamiana* leaves were inoculated with wild‐type TbCSV or TbCSV_dC3_ indivudually followed by agroinfiltration with a binary vector expressing TbCSV C3 (pCV‐C3) or an empty vector (pCV) at 2 dpi. At 3 days post‐agroinfiltration (dpai), the over‐accumulation of the *C3* transcript in TbCSV_dC3_/pCV‐C3 infiltrated leaves was confirmed by RT‐qPCR (Figure 3a). Compared to TbCSV/pCV control, qPCR analysis showed a decrease in viral DNA accumulation in TbCSV_dC3_/pCV infiltrated leaves by approximately 60%, indicating impaired replication activity of TbCSV_dC3_. Notably, co‐infiltration of TbCSV_dC3_ with pCV‐C3 restored viral DNA accumulation levels comparable to the TbCSV/pCV controls (Figure 3b). These results indicate that deletion of the 3′ end 144 nucleotides of TbCSV *C3* impedes its REn activity, which can be complemented in trans by heterogeneously expressed C3 protein.

Our findings suggest that the 3′ end 144 nucleotides of *C3* (amino acids 88–134) are needed for its replication enhancement activity. To further precisely delineate this critical region, a series of C3 truncated mutants expressed by the pCV vector, including C3(1–87) (reserved the amino acid region from residues 1 to 87 of C3), C3(1–94), C3(1–101), C3(1–108), C3(1–115), C3(1–122) and C3(1–129), were constructed and subjected to local infection assays accompanied by TbCSV_dC3_ in *N. benthamiana*, as described earlier. The infiltrations of TbCSV_dC3_/C3 and TbCSV_dC3_/pCV empty vector served as positive and negative controls, respectively. At 3 dpai with pCV‐C3 or pCV‐C3 mutants, the accumulation levels of viral DNA in infiltrated areas were determined by qPCR, and our results showed that only the C3(1–129) mutant successfully restored TbCSV_dC3_ accumulation to levels similar to the positive control (Figure 3c). In contrast, none of the other C3 mutants showed significant increases in viral DNA accumulation, as shown in TbCSV_dC3_/pCV negative control (Figure 3c). The 35S promoter‐mediated transcription of the *C3* gene and all the *C3* mutants was confirmed by RT‐PCR (Figure S2a). Additionally, we examined the function of C3 mutants in the context of TbCSV_∆C3_ infection, a mutant virus with untranslated *C3* that was constructed and used in our previous report (Sun et al. 2020). Two C3 mutants, C3(1–122) and C3(1–129), were subjected to local infection analyses as mentioned earlier, and the results further validated the functional complementation of C3(1–129) to TbCSV_∆C3_ replication (Figure S3).

Next, co‐infection assays with TbCSV_dC3_ and PVX vector expressing each of the C3 truncated mutants were performed, and the combinations TbCSV_dC3_/C3 and TbCSV_dC3_/PVX served as positive and negative controls, respectively. As shown in Figure 3d, at 7 dpi, only co‐infection with TbCSV_dC3_ and C3(1–129) induced an obvious downward curling phenotype on upper systemically infected leaves, mimicking the symptoms seen in positive control plants. In contrast, no visible abnormal growth phenotype was observed in other C3 truncated mutants or negative control *N. benthamiana* plants. Correspondingly, among all the C3 mutants, co‐infection with PVX‐C3(1–129) exclusively restored the viral DNA accumulation of TbCSV_dC3_ in upper new leaves of infected plants, similar to full‐length *C3*‐expressed positive control (Figure 3e). The transcription of the *C3* gene and all the *C3* mutants was confirmed by RT‐PCR (Figure S2b). Together, these results suggest that the C‐terminal amino acid region (residues 123–129) of C3 is critical for its replication enhancement activity.

### 2.4 The Amino Acid Motif (residues 123–129) of C3 Is Required for Effective Infection of TbCSV

**FIGURE 4:**
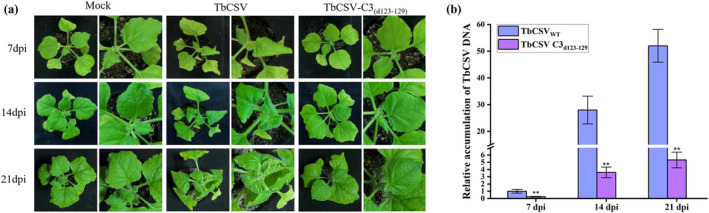
The 123–129 amino acid residues of TbCSV C3 are required for viral DNA accumulation and symptoms development. (a) Symptoms of *Nicotiana benthamiana* plants infected with wild‐type TbCSV and TbCSV_(d123–129)_ mutant infectious clone at 7, 14 and 21 days post‐inoculation (dpi). Mock‐inoculated *N. benthamiana* plants served as controls. (b) Relative accumulation of TbCSV DNA. *N. benthamiana* 25S ribosomal RNA was used as the internal reference. The results were reproduced in three independent experiments using 12 plants per treatment. Error bars represent means ± SEM, and significant differences were indicated using Student's *t* test, **p < 0.01. These experiments were performed with three independent biological replicates with similar results.

To further determine the biological significance of the amino acid motif (residues 123–129) of C3 in TbCSV infection, we constructed a mutant infectious clone of TbCSV by deleting the coding sequence of the amino acids 123–129 of C3 from the viral genome (designated as TbCSV‐C3_(d123–129)_). Subsequently, TbCSV and TbCSV‐C3_(d123–129)_ were agroinoculated into *N. benthamiana* plants individually, with buffer inoculation (Mock) serving as a control. Symptoms and viral genomic DNA accumulation were monitored at 7, 14 and 21 dpi. As expected, typical and gradually aggravating symptoms, including leaf curling and shrinking, were observed in TbCSV‐infected plants. In contrast, TbCSV‐C3_(d123–129)_ infection only induced mild downward curling of the upper new leaves (Figure 4a). Correspondingly, qPCR analysis confirmed significantly lower viral accumulation of TbCSV‐C3_(d123–129)_ compared to wild‐type TbCSV in the systemically infected leaves (Figure 4b). Together, these results suggest that the amino acid motif from residues 123 to 129 of C3 is crucial for facilitating viral accumulation and infection.

### 2.5 The Amino Acid Motif (residues 123–129) of C3 Is Required for Its Interaction With DNA Polymerase α

To uncover the mechanism underlying the biological importance of the C‐terminal amino acid motif of C3, we investigated whether this region is involved in its functional characteristics, including the subcellular localisation and intra‐ and intermolecular interactions. Given that geminiviruses replicate in the nuclei of host cells, the nuclear localisation of the C3 protein is considered essential for its replication enhancement activity. Thus, using agroinfiltration‐mediated transient expression assays, we investigated the subcellular localisation of C3 and a C3 truncated mutant (1–122 amino acids [aa]), which expressed the amino acid residues from 1 to 122. Our results showed that C3‐GFP exclusively localised in the nuclei of *N. benthamiana* leaf cells, and interestingly, deletion of the C‐terminal region (residues 123–134) of C3 did not alter its subcellular localisation (Figure S4).

**FIGURE 5:**
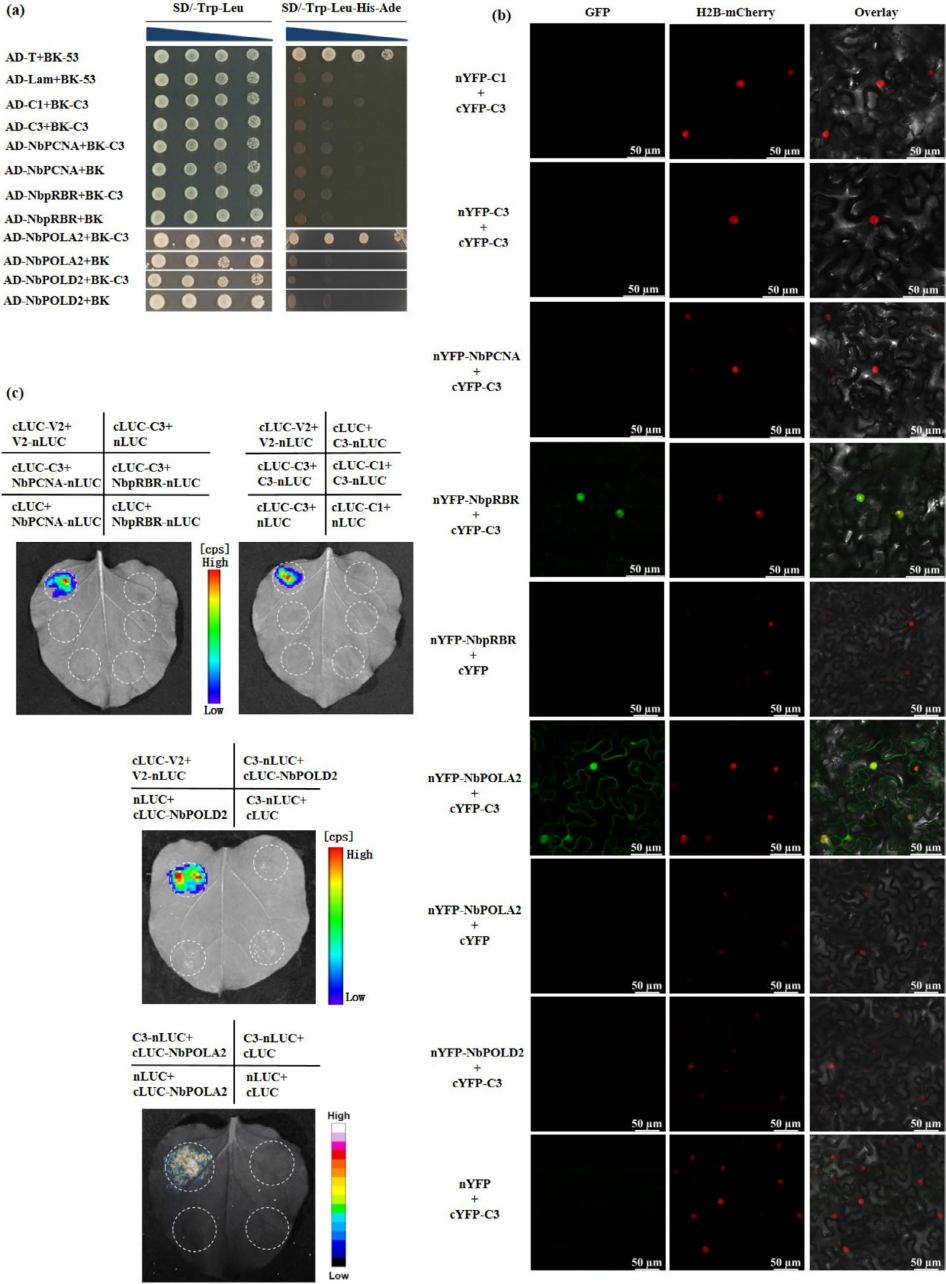
Interactions between TbCSV C3 and NbPOLA2 in yeast and in planta. (a) Yeast two‐hybrid (Y2H) analysis of intra‐ and intermolecular interactions of TbCSV C3. Recombinant plasmid pairs as that indicated were co‐transformed into yeast strain Y2H‐Gold. Serial dilutions (10‐fold) of transformants were spotted on SD/−Trp−Leu and SD/‐Trp−Leu−His−Ade selective media. Co‐transformation with AD‐T+BK‐53 served as a positive control. Yeast co‐transformed with AD‐Lam+BK‐53, AD‐NbPCNA+BK, AD‐NbpRBR+BK, AD‐NbPOLA2+BK and AD‐NbPOLD2+BK were negative controls. Images were taken after 72 h. (b) Bimolecular fluorescence complementation (BiFC) analysis of the intra‐ and intermolecular interactions of TbCSV C3 in planta. Leaves co‐infiltrated with cYFP‐C3 and one of the candidates including nYFP‐C3, ‐C1, ‐NbPCNA, ‐NbpRBR, ‐NbPOLA2 and ‐NbPOLD2 were examined under confocal microscope. Co‐infiltration with cYFP‐C3+nYFP, cYFP+nYFP‐NbPOLA2 and cYFP+nYFP‐NbpRBR served as negative controls. Histone 2B‐RFP (H2B‐RFP) was used as a nuclear marker. (c) Luciferase complementation imaging (LCI) analysis of the interaction between C3 and candidate proteins in *Nicotiana benthamiana* leaves. Leaves were co‐infiltrated with *Agrobacterium* strains harbouring the indicated plasmids and subjected to fluorescence detection after 3 days. Co‐infiltration with V2‐nLUC and cLUC‐V2 served as a positive control, while co‐infiltration combinations nLUC+cLUC, C3‐nLUC+cLUC, nLUC+cLUC‐C3, nLUC+cLUC‐C1, NbpRBR‐nLUC+cLUC, NbPCNA‐nLUC+cLUC, nLUC+cLUC‐NbPOLA2 and nLUC+cLUC‐NbPOLD2 served as negative controls.

Previous research on begomoviruses has shown that the oligomerisation of C3/AC3, hetero‐oligomerisation with C1/AC1 (AL1) and interactions with several host factors such as PCNA, pRBR and DNA polymerases α and δ are important for its replication enhancement activity (Settlage et al. 2001; Settlage et al. 1996; Settlage et al. 2005; Castillo et al. 2003; Wu et al. 2021). We hypothesised that the C‐terminal region of the C3 protein might mediate its interactions with both viral and host proteins. To test this, we first examined the self‐interaction of TbCSV C3 protein, as well as its interaction with TbCSV C1, NbPCNA, NbpRBR, NbPOLA2 and NbPOLD2 proteins by yeast two‐hybrid (Y2H) assays. Our results showed that only the yeast transformants with BK‐C3 and AD‐NbPOLA2 were able to grow on the selective medium SD/−Trp−Leu−His−Ade (Figure 5a). The positive interactions of TYLCV C3 with these factors in yeast cells suggest their proper expressions (Figure S5). The protein interactions were further investigated by in planta bimolecular fluorescence complementation (BiFC) and luciferase complementation imaging (LCI) assays. The TbCSV‐encoded V2–V2 protein combination served as a positive control, whose self‐interaction was verified in our previous report (Li, Li, et al. 2021). Of note, in BiFC experiments, the reconstituted fluorescence could be observed in C3‐NbPOLA2 or C3‐NbpRBR combinations expressed in leaf cells (Figures 5b and S6), whereas only the interaction between C3 and NbPOLA2 could be confirmed by the LCI assay (Figure 5c). These results indicate that NbPOLA2, an important component of the host cellular DNA replication machinery, stably interacts with TbCSV C3 in yeast and in planta.

**FIGURE 6:**
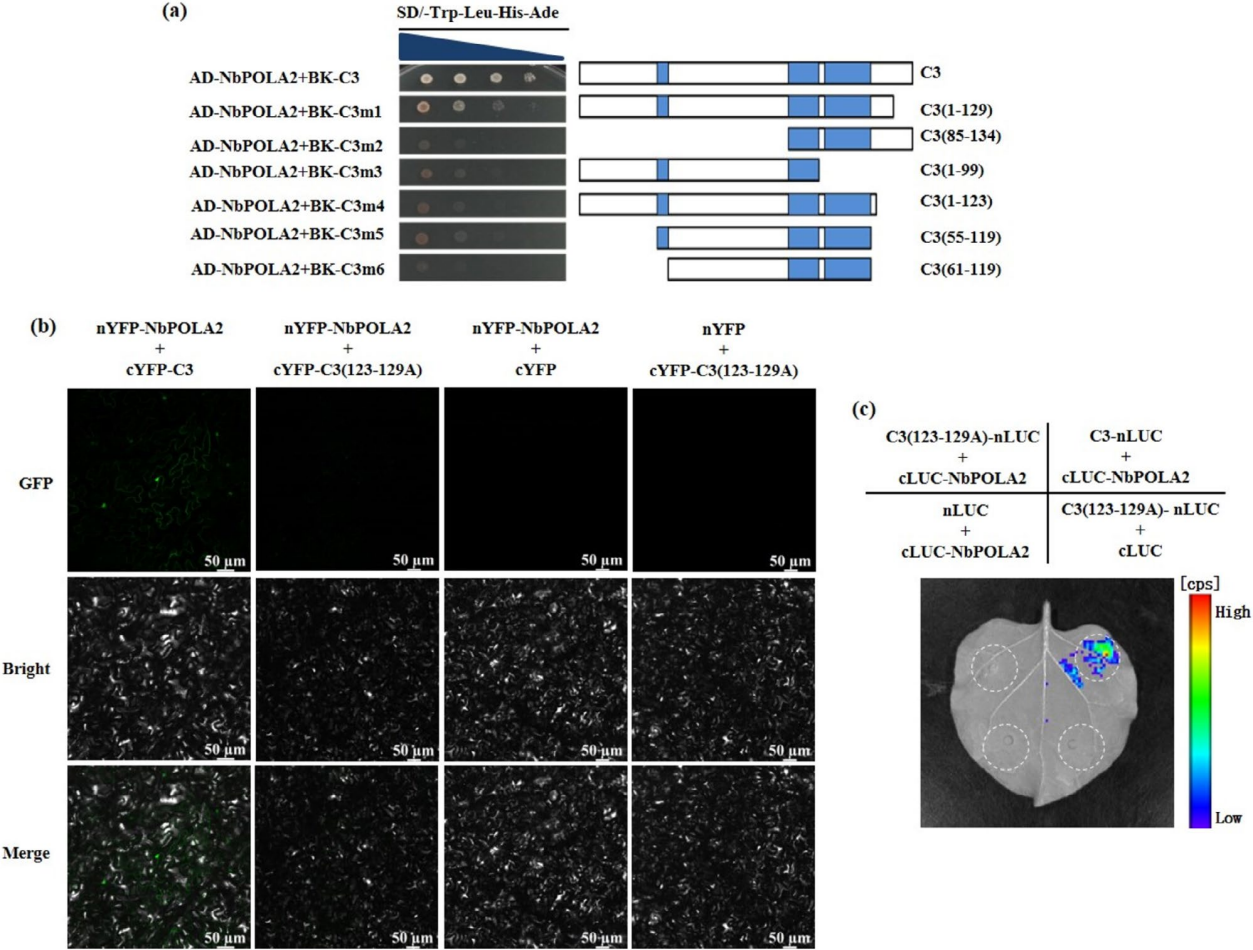
Interaction analysis between NbPOLA2 and C3 mutants. (a) Yeast two‐hybrid (Y2H) analysis of the amino acid region in TbCSV C3 required for interaction with NbPOLA2. The schematic diagram indicates the truncated mutants of C3. Transformants were serially diluted 10‐fold and spotted on SD/−Trp−Leu−His−Ade selective medium. Images were captured after 72 h. (b) Bimolecular fluorescence complementation (BiFC) analysis of the interaction between NbPOLA2 and the C3(123–129A) mutant. Fluorescence in *Nicotiana benthamiana* leaves co‐infiltrated with nYFP‐NbPOLA2 and cYFP‐C3(123–129A), in which the amino acids from residues 123 to 129 were substituted with seven alanines, was examined using a confocal microscope. Co‐infiltration with nYFP‐NbPOLA2+cYFP and nYFP+cYFP‐C3(123–129A) served as negative controls, while co‐infiltration of nYFP‐NbPOLA2 with cYFP‐C3 was used as a positive control. (c) Luciferase complementation imaging (LCI) analysis of the interaction between NbPOLA2 and the C3(123–129A) mutant in *N. benthamiana*. *Agrobacterium* strains harbouring C3(123–129A)‐nLUC and cLUC‐NbPOLA2 were co‐infiltrated into *N. benthamiana* leaves, with luciferase detection performed 3 days post‐infiltration. Co‐infiltration of C3‐nLUC with cLUC‐NbPOLA2 served as a positive control, while nLUC+cLUC‐NbPOLA2 and C3(123–129A)‐nLUC+cLUC were used as negative controls.

Considering the importance of the amino acid motif (residues 123–129) of C3 for its replication enhancement activity, it is intriguing to know whether this region is critical for its interaction with NbPOLA2. Interestingly, the Y2H analyses showed that yeast transformants harbouring BK‐C3m1 (reserving the amino acid residues 1–129 of C3) and NbPOLA2 could grow on selection medium, while the mutant BK‐C3m4 lacking the amino acids from 124 to 134 failed to interact with NbPOLA2 (Figure 6a). It is worth mentioning that the motif (residues 123–129) of the C3 protein is essential but not sufficient to mediate its interaction with NbPOLA2, because the C3(85–134) mutant failed to interact with NbPOLA2 in yeast (Figure 6a). To further clarify the biological importance of this seven‐amino acid motif of C3 in its interaction with NbPOLA2, a C3 mutant in which the amino acids from 123 to 129 were substituted with seven alanines (C3(123–129A)) was constructed and subjected to BiFC and LCI assays. Notably, in both assays, no visible fluorescence or luciferase signal was detected in leaf tissue co‐expressing NbPOLA2 and C3(123–129A), suggesting the abolishment of protein interaction (Figures 6b,c and S7). Taken together, the seven‐amino acid motif from residues 123 to 129 of C3 is essential for its interaction with NbPOLA2.

### 2.6 NbPOLA2 Positively Regulates Systemic Infection of TbCSV

**FIGURE 7:**
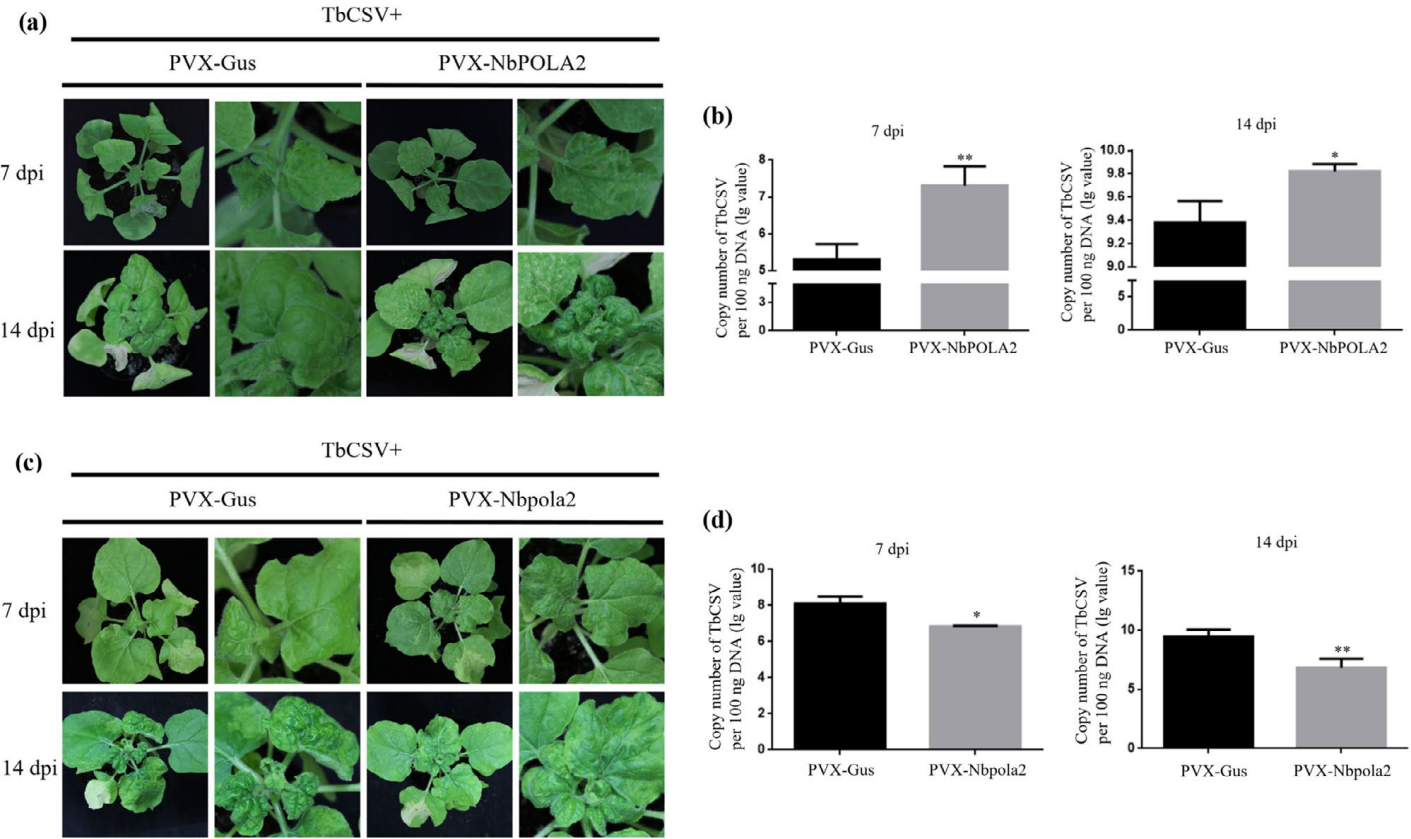
*NbPOLA2* positively regulates TbCSV infection. (a, b) Overexpression of *NbPOLA2* facilitates TbCSV infection. (a) Symptoms of *Nicotiana benthamiana* plants infected with PVX‐NbPOLA2 or the PVX‐GUS control at 7 days post‐inoculation (dpi). (b) Quantitative PCR (qPCR) analysis of TbCSV DNA accumulation at 7 and 14 dpi. (c, d) Silencing of *NbPOLA2* inhibits TbCSV infection. (c) Symptoms of *N. benthamiana* plants infected with PVX‐Nbpola2 or PVX‐GUS at 7 and 14 dpi. (d) qPCR analysis of TbCSV DNA accumulation at 7 and 14 dpi. In panels (b) and (d), *N. benthamiana* 25S ribosomal RNA was used as the internal reference. Each experiment was conducted independently three times with four plants for each treatment each time. Error bars represent means ± SEM, and significant differences were determined using Student's *t* test, *p < 0.05, **p < 0.01. These experiments were performed with three independent biological replicates with similar results.

The biological significance of the seven‐amino acid motif (residues 123–129) of C3 in its replication enhancement activity and its interaction with NbPOLA2 prompted us to investigate the effects of NbPOLA2 on TbCSV infection, by virus‐induced gene silencing (VIGS) and overexpression assays. First, we overexpressed NbPOLA2 in *N. benthamiana* plants using a PVX vector; PVX‐GUS, harbouring the partial sequence of β‐glucuronidase (*gus*) gene, served as a control. By 7 dpi, RT‐qPCR analysis confirmed that the relative expression level of *NbPOLA2* in PVX‐NbPOLA2‐inoculated plants was increased by approximately 300‐fold compared to that in PVX‐GUS‐inoculated plants, while no obvious growth defects were observed in *NbPOLA2*‐overexpressing *N. benthamiana* plants (Figure S8a,b). Subsequently, the effect of overexpressing *NbPOLA2* on TbCSV infection was investigated. PVX‐NbPOLA2‐ and PVX‐GUS‐infected *N. benthamiana* plants were inoculated with TbCSV at 5 dpi. At 7 dpi with TbCSV, PVX‐NbPOLA2‐inoculated plants exhibited a more pronounced downward curling phenotype of newly emerging leaves compared to controls (Figure 7a). At 14 days after TbCSV inoculation, PVX‐NbPOLA2‐infected plants showed more severe leaf curling and shrinking symptoms. qPCR analysis revealed that the accumulation of TbCSV DNA in *NbPOLA2*‐overexpressing plants was significantly higher at both 7 and 14 dpi compared to control plants (Figure 7b). These results indicate that the overexpression of *NbPOLA2* facilitates TbCSV infection.

Next, we examined the effects of silencing *NbPOLA2* on TbCSV infection. Because silencing of *NbPOLA2* in *N. benthamiana* plants using the tobacco rattle virus (TRV)‐based VIGS system caused an intense local and systemic necrosis phenotype, the PVX vector was employed to perform the *NbPOLA2* gene silencing assay. To this end, a partial nucleotide sequence of 300 bp in length from *NbPOLA2* was cloned into the PVX vector to generate the PVX‐Nbpola2 construct for the VIGS assay. At 7 days post‐inoculation with PVX‐Nbpola2, the expression of *NbPOLA2* was down‐regulated by approximately 60% compared with PVX‐GUS‐inoculated control plants (Figure S8e). At 14 dpi, the PVX‐Nbpola2‐inoculated *N. benthamiana* plants exhibited leaf shrinking and dwarf phenotype, while the cell death was not induced (Figure S8c,d). When co‐infected with TbCSV, the PVX‐Nbpola2 and TbCSV co‐infected *N. benthamiana* plants showed milder leaf curling and shrinking symptoms compared to PVX‐GUS and TbCSV co‐infected control plants at 7 and 14 dpi with TbCSV (Figure 7c). As expected, the viral titres of TbCSV in *NbPOLA2*‐silenced plants were obviously lower than those in control plants at both 7 and 14 dpi (Figure 7d). Taken together, these findings indicate that *NbPOLA2* positively regulates TbCSV infection and viral accumulation.

### 2.7 Overexpression of *NbPOLA2* Enhances TbCSV Replication

**FIGURE 8:**
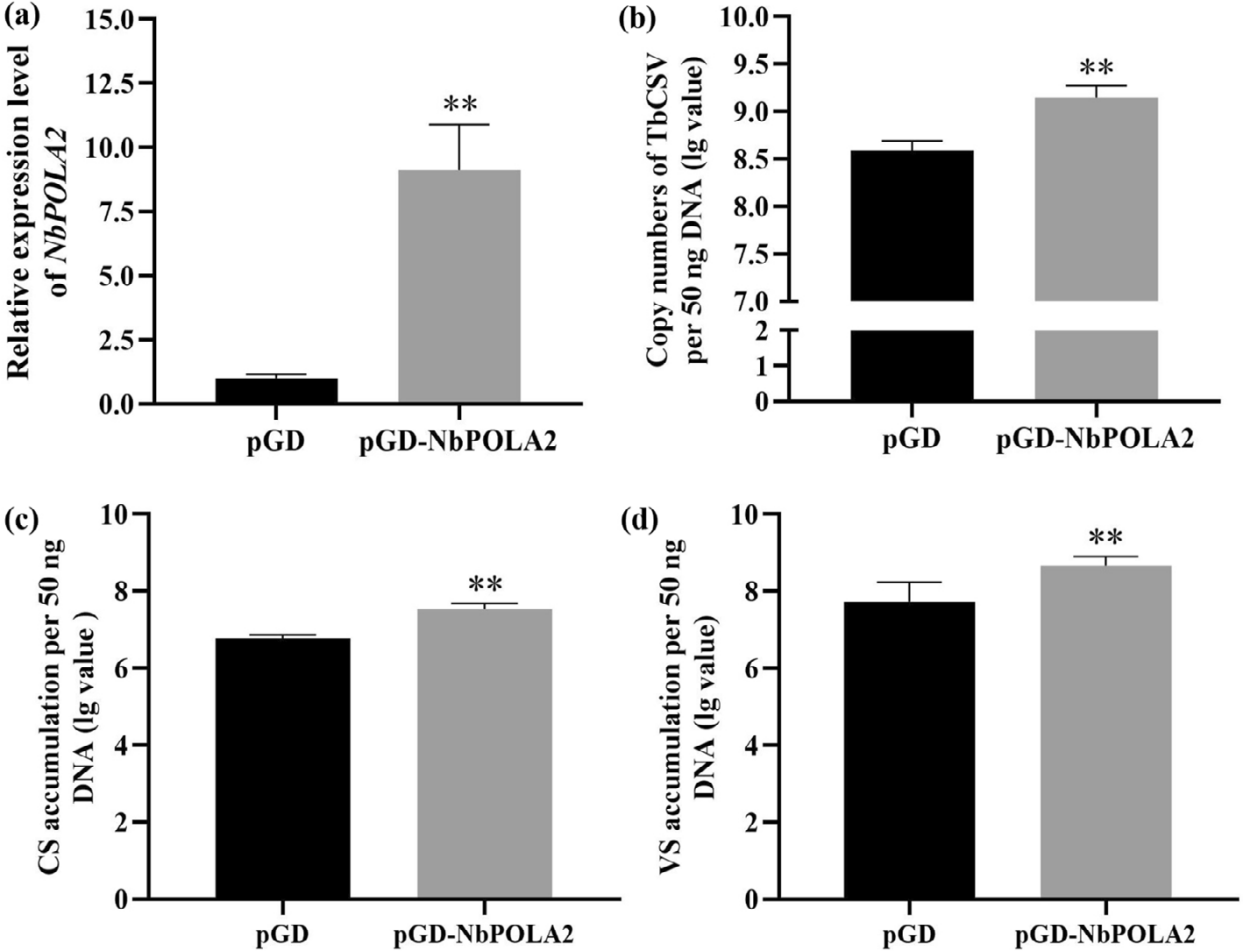
*NbPOLA2* enhances TbCSV replication. In local infection assays, pGD‐NbPOLA2 or pGD empty vector was infiltrated into *Nicotiana benthamiana* leaves pre‐inoculated with TbCSV for 2 days, and the leaf samples were collected at 3 days post‐agroinfiltration. (a) Reverse transcription quantitative PCR (RT‐qPCR) analysis of *NbPOLA2* expression levels in pGD‐NbPOLA2 and pGD‐infiltrated leaves. *NbActin* was used as the internal reference. (b) qPCR analysis of TbCSV DNA accumulations in pGD‐NbPOLA2 and pGD‐infiltrated leaves. (c, d) Quantifications of the viral complementary strand (CS) (c) and viral strand (VS) (d) in *N. benthamiana* plants overexpressing *NbPOLA2* (pGD‐NbPOLA2) or the control (pGD). In panels (b) to (d), *N. benthamiana* 25S ribosomal RNA was used as the internal reference. Data represent the mean of five independent biological replicates. Error bars represent means ± SEM. Significant differences were determined using Student's *t* test, **p < 0.01.

In the DNA replication machinery of eukaryotes, POLA2 protein is a regulatory subunit of DNA polymerase α, which is responsible for priming DNA replication (Jain et al. 2018). To investigate the role of NbPOLA2 in TbCSV replication, the local infection assays were performed as described earlier. TbCSV was used to pre‐inoculate *N. benthamiana* plants, and then the *Agrobacterium* cells carrying pCV‐NbPOLA2 were infiltrated into the inoculated leaves to transiently overexpress *NbPOLA2* at 2 dpi with TbCSV. The *N. benthamiana* leaves pre‐inoculated with TbCSV followed by pCV‐GFP infiltration served as controls. At 3 dpai with pCV‐NbPOLA2 or pCV‐GFP, the infiltrated leaves were harvested for RNA and DNA extraction. RT‐qPCR analyses confirmed that the expression level of *NbPOLA2* was elevated approximately eightfold in pCV‐NbPOLA2‐infiltrated leaves compared to control leaves, and qPCR results showed that the TbCSV DNA in *NbPOLA2*‐overexpressing leaves accumulated significantly higher than that in controls (Figure 8a,b).

To explore the mechanisms by which *NbPOLA2* facilitates viral replication of TbCSV, a two‐step anchored qPCR was conducted to measure the accumulation of viral ssDNA (viral strand, VS) and the dsDNA intermediate (presented by complementary strand, CS) in a local infection assay (Rodríguez‐Negrete et al. 2014; Wu et al. 2021). Interestingly, overexpression of *NbPOLA2* significantly increased the complementary strand accumulation, hence facilitating the subsequent production of the viral strand (Figure 8c,d). Our findings suggest that NbPOLA2 may regulate the initial synthesis of the viral complementary strand and the subsequent generation of the double‐stranded DNA intermediate during TbCSV infection, ultimately enhancing viral accumulation.

## 3 Discussion

**FIGURE 9:**
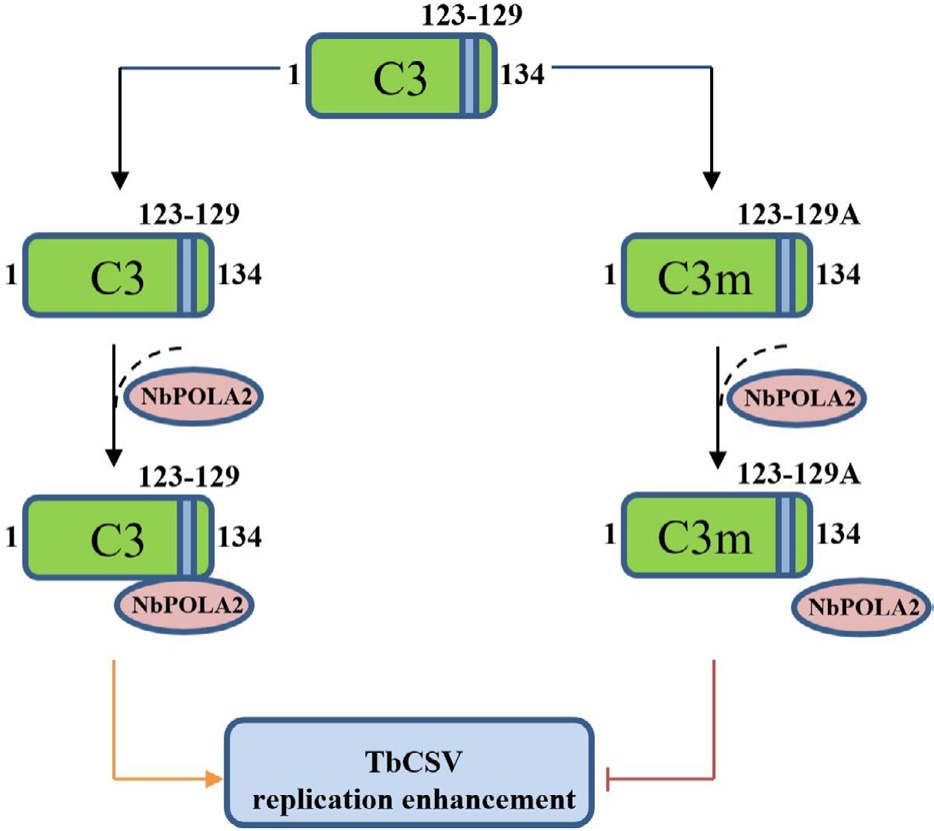
Proposed working model for the seven‐amino acid motif‐dependent viral replication enhancement activity of TbCSV C3. A C‐terminal seven‐amino acid motif spanning from residues 123 to 129 of the C3 protein is responsible for recruiting the host factor NbPOLA2 to facilitate viral replication. Mutation of the 123–129 motif to seven alanines abolishes the interaction between C3 and NbPOLA2, hence impairing the replication enhancement activity of C3.

The C3/AC3 protein of geminiviruses functions as a replication enhancer to increase geminiviral DNA accumulation levels (Fondong 2013; Settlage et al. 2005; Sung and Coutts 1995a, 1995b; Sunter et al. 1990). Our previous study revealed that mutations in the start codon of the TbCSV C3 protein mildly attenuated symptom development and reduced viral accumulation up to 15 dpi, while the mutant virus accumulated to a similar level as the wild‐type TbCSV at 20 dpi (Sun et al. 2020). In this study, we showed that deletion or replacement of the 3′ end region of the *C3* gene that does not overlap with *C2* sharply decreased viral accumulation and symptom development (Figure 1). We further found that the seven‐amino acid motif from residues 123 to 129 of C3 is crucial for its interaction with NbPOLA2, which is a component of the host DNA replication machinery and positively regulates TbCSV replication and infection. Based on these findings, a working model of the viral replication enhancement by TbCSV C3 was proposed. In this model, C3 can exert its REn activity by recruiting NbPOLA2, a positive regulator of TbCSV replication. Mutation of the seven‐amino acid motif (residues 123–129) of C3 abolishes its interaction with NbPOLA2 and leads to the impairment of its replication enhancement activity (Figure 9).

Our findings are consistent with studies on other geminiviruses like TGMV and African cassava mosaic virus (ACMV), where disrupting AC3 expression by deletion or insertion of nucleotide sequences markedly affected symptom development and viral DNA accumulation (Elmer et al. 1988; Morris et al. 1991). However, why does the deletion of the 3′ end region of *C3* has more pronounced effects than mutations in its start codon on TbCSV infection? Firstly, the truncated mutation of *C3* may change the genomic structure of TbCSV. An increasing body of evidence suggests that the structural motifs within viral nucleotide sequences generally play roles in viral replication, transcription, translation, genome packaging and even the regulation of host antiviral defences (Hefferon et al. 2006; Shen and Miller 2004; Wang, Yang, et al. 2022; Yan et al. 2022; Stockley et al. 2013; Wikström et al. 2007). For geminiviruses, the hairpin motif in the intergenic region is a well‐established element essential for the geminivirus replication origin (Orozco and Hanley‐Bowdoin 1996; Fontes et al. 1994; Orozco et al. 1998). Interestingly, a report identified a conserved and biologically functional stem‐loop structure in the genomes of TYLCV, East African cassava mosaic virus (EACMV) and Malvastrum yellow vein Yunnan virus (MYVYV), derived from a sequence that covered the 3′ end region of *C3*/*AC3* (Muhire et al. 2014). Thus, deleting or replacing the 3′ end region of the *C3* gene may disrupt the critical secondary structures necessary for viral infectivity and DNA accumulation (Figure S9). However, it is worth noting that the infectivity and viral accumulation of the TbCSV_dC3_ mutant could be restored when *C3* was overexpressed in trans, suggesting that a high expression level of *C3* can compensate for the secondary structure changes (Figure 2). Secondly, as presented in our work, deletion of the C‐terminal amino acid motif of TbCSV C3 abolished its interaction with NbPOLA2, a component of plant DNA replication machinery that is recruited by TbCSV to support viral genomic replication. Meanwhile, whether the truncated C3 mutant can induce host antiviral defence through interacting with host factors needs to be further investigated.

Intra‐ and intermolecular interactions have been reported to be crucial for the replication enhancement activity of C3/AC3 protein (Settlage et al. 2005; Pasumarthy et al. 2010). For instance, the homo‐oligomerisation of TYLCV C3 protein and its hetero‐oligomerisation with C1 are required to enhance genomic DNA accumulation (Settlage et al. 2005). Geminiviral C3/AC3 protein also recruits host factors to facilitate virus replication. The interactions of C3 encoded by TYLCV and tomato yellow leaf curl Sardinia virus (TYLCSV) with proliferating cell nuclear antigen (PCNA), a regulator of the host cell cycle, have been reported to be important for the replication enhancement activity of C3 (Castillo et al. 2003; Settlage et al. 2005). Recently, two components of the host cellular DNA replication machinery, the subunits of DNA polymerase α (POLA2) and DNA polymerase δ (POLD2) were verified to be directly recruited by TYLCV C3 protein (Wu et al. 2021). In this study, we analysed the interactions of TbCSV C3 with viral and host proteins. Among all of the tested proteins, only NbPOLA2 interacted with TbCSV C3, which might be because of the high variation of different C3/AC3 proteins (Figure S10). POLA2 is a subunit of the DNA polymerase α complex, and our results showed that NbPOLA2 may function in the generation of the dsDNA intermediate during TbCSV replication (Figure 8). Additionally, transcription levels of the other subunits in the DNA polymerase α complex were determined, and the RT‐qPCR results revealed significantly higher accumulation of the transcriptions of *NbPOLA1.1/1.2*, *NbPOLA3.1/3.2* and *NbPOLA4.2* in *NbPOLA2*‐overexpressing leaves, suggesting some other pleiotropic effects of *NbPOLA2* on plants (Figure S11).

In summary, we revealed in this study that the seven‐amino acid motif (residues 123–129) in TbCSV C3 is essential for functioning in viral replication enhancement, through recruiting NbPOLA2 to facilitate the viral dsDNA replication intermediate synthesis. Our results deepen our understanding of the mechanism underlying TbCSV C3‐mediated replication enhancement and provide potential insights for the development of strategies to control viral diseases caused by TbCSV.

## 4 Experimental Procedures

### 4.1 Plant Materials and Growth Conditions

Wild‐type *N. benthamiana* plants were cultivated in a growth room at 24°C under a 16‐h/8‐h (light/dark) photoperiod cycle.

### 4.2 Plasmid Construction

To obtain TbCSV *C3* truncated mutant infections, a clone in which the 3′‐terminal region of the C3 open reading frame (ORF) that does not overlap with the C2‐encoding sequence was deleted, a pGEM‐T vector derived construct harbouring 1.0 copy of the TbCSV clone (isolate Y35, GenBank No. AJ420318) was used as a template for PCR. Firstly, overlap extension PCR was performed using two primer pairs, dC3‐R/Y35b‐F and dC3‐F/Y35b‐R, to delete the 3′ end region of the *C3* gene. Then, the cleaned PCR product was inserted into the pGEM‐T vector to get one copy of a mutant TbCSV clone (pGEM‐Y35A_dC3_). Next, the 0.9 copy of Y35A_dC3_ was released from the pGEM‐Y35A_dC3_ plasmid with enzymes BamHI‐HindIII and inserted into the binary expression vector pBINPLUS to produce pBINPLUS‐0.9A_dC3_. Finally, a 1.0 copy of Y35A_dC3_ was digested from pGEM‐T‐Y35A_dC3_ with the enzyme BamHI and inserted into pBINPLUS‐0.9A_dC3_ to generate the infectious 1.9 copy of Y35A_dC3_ (referred to as TbCSV_dC3_).

To obtain TbCSV mutant infections, a clone in which the 3′ terminal region of C3 was replaced by the *iLOV* gene sequence, overlap extension PCR was performed using three primer pairs dC3‐iLOV‐R/Y35b‐F, iLOV‐F/iLOV‐R and dC3‐iLOV‐F/Y35b‐R to replace the 3′ end region of the *C3* gene with the *iLOV* gene sequence, with the pGEM‐Y35A plasmid as the template. Subsequently, the cleaned PCR product was inserted into pGEM‐T to get 1.0 copy of a mutant TbCSV clone (pGEM‐Y35A_dC3‐iLOV_). Then, the 0.9 copy and 1.0 copy of Y35A_dC3‐iLOV_ were released from the pGEM‐Y35A_dC3‐iLOV_ plasmid using the method presented earlier. Finally, the 1.0 copy of Y35A_dC3‐iLOV_ was inserted into pBINPLUS‐0.9A_dC3‐iLOV_ to generate the infectious 1.9 copy of Y35A_dC3‐iLOV_ (referred to as TbCSV_dC3‐iLOV_).

To obtain TbCSV mutant infections clone in which the amino acids 123–129 were deleted (TbCSV‐C3_(d123–129)_), the primer pairs C3_(d123–129)_‐R/Y35b‐F and C3_(d123–129)_ ‐F/Y35b‐R were used to perform overlap extension PCR, and the other procedures are the same as those used for generating TbCSV_dC3_ mutant.

To identify the critical region of C3 which is required for the replication enhancement activity, the coding sequence of C3 and a series of C3 mutants were amplified from TbCSV‐Y35 isolate infectious clone and inserted into BamHI‐SacI sites of a transient expression vector pCV‐GFP‐N1 and into ClaI‐SalI sites of a PVX vector pGR106.

For Y2H assays, the TbCSV *C3* gene sequence and *C3* deletion derivatives were amplified and cloned into the pGBK‐T7 vector to produce BK‐C3, BK‐C3m1, BK‐C3m2, BK‐C3m3, BK‐C3m4, BK‐C3m5 and BK‐C3m6. The coding sequences of TbCSV C1 and C3, NbPCNA, NbpRBR, NbPOLA2 and NbPOLD2 were amplified and cloned into the pGAD‐T7 vector.

For BiFC assays, the coding sequence of TbCSV C3 was amplified and cloned into the pCV‐cYFP vector to generate cYFP‐C3. The full‐length sequences of TbCSV C1 and C3, NbPCNA, NbRBR, NbPOLA2 and NbPOLD2 were amplified and inserted into the pCV‐nYFP vector individually.

For the LCI assays, the full‐length sequences of TbCSV C3, NbPCNA and NbpRBR were cloned into the pCAMBIA‐NLuc vector individually. Meanwhile, the coding sequences of C3, C1, NbPOLA2 and NbPOLD2 were cloned into the pCAMBIA‐CLuc vector individual (Chen et al. 2008).

To overexpress and silence *NbPOLA2*, the full‐length sequence and a fragment of *NbPOLA2* were amplified and cloned into the pGR106 vector to generate PVX‐NbPOLA2 and PVX‐Nbpola2, respectively.

### 4.3 *Agrobacterium*‐Mediated Transient Expression

*Agrobacterium*‐mediated transient expression assay was performed as described previously (Li et al. 2018). In brief, *Agrobacterium tumefaciens* GV3101 harbouring the recombinant plasmids was cultured in Luria Bertani (LB) broth with appropriate antibiotics for approximately 16 h at 28°C, and then the cultures were centrifuged at 4000 *g* for 10 min and resuspended in MMA buffer (10 mM MgCl_2_, 10 mM MES, pH 5.7, 100 μM acetosyringone) to OD_600_ = 0.5. After 3 h at room temperature in the dark, the resuspension solution was injected into the fully expanded leaves of 4‐week‐old *N. benthamiana* plants using a 1‐mL needleless syringe.

### 4.4 Local and Systemic Infection Assays

*Agrobacterium*‐mediated virus inoculation was used for local and systemic infection assays. *A. tumefaciens* EHA105 harbouring the viral infectious clones was incubated in LB broth with appropriate antibiotics overnight at 28°C and resuspended in MMA buffer to OD_600_ = 1.0 after centrifugation (4000 *g*, 10 min). After 3 h at room temperature in the dark, the resuspension solution was used for inoculation. The local infection assay was performed as previously described for TYLCV inoculation, with minor modifications (Wu et al. 2021). In detail, the inoculation solution was infiltrated into the fully expanded leaves of 4‐week‐old *N. benthamiana* plants. At 2 dpi, *A. tumefaciens* GV3101 carrying the binary expression vector for protein overexpression was infiltrated into the TbCSV pre‐inoculated leaves through the agroinfiltration method as presented earlier. After 3 days, the inoculated leaves were harvested for further analyses. For systemic infection of TbCSV, the resuspension solution carrying the virus was inoculated into the leaves of 4‐ to 6‐leaf stage *N. benthamiana* plants through the agroinfiltration method.

### 4.5 Y2H Assay

Y2H assays were conducted using the yeast strain Y2H‐Gold according to the manufacturer's instructions (Clontech). The transformants were grown on double dropout medium (SD/−Trp−Leu) with agar for 72 h, and then the colonies were picked and cultured on the quadruple dropout medium (SD/−Trp−Leu−His−Ade) with agar after gradient dilution.

### 4.6 BiFC Assay

The BiFC assays were conducted as described previously (Li, Guo, et al. 2022). The plasmids were transformed into *A. tumefaciens* GV3101. The cultured *Agrobacterium* cells were resuspended in MMA buffer to an OD_600_ of 0.5, and then the YFP split combinations were subjected to co‐infiltration. At 3 dpai, the fluorescence was detected using a confocal laser scanning microscope (LSM780; Zeiss) at a wavelength of 488 nm.

### 4.7 LCI Assay

LCI assays were performed as described previously (Chen et al. 2008). The combinations were co‐agroinfiltrated into the leaves of *N. benthamiana* plants. At 3 dpi, the leaves were detached and sprayed with 1 mM luciferin (Invitrogen). The luciferase activity was detected using a low‐light cooled CCD imaging apparatus (iXon, Andor Technology), and the pictures were taken after 15 min exposure.

### 4.8 RT‐qPCR Analysis

Total RNA was extracted from *N. benthamiana* leaves using the RNAiso Plus reagent (TaKaRa). The first‐strand complementary DNA was synthesised, and the host genomic DNA was removed using an RT reagent kit (TaKaRa). qPCR was conducted using the NovoStart SYBR qPCR SuperMix Plus kit (Novoprotein). All the operations were according to the manufacturer's instructions. Relative accumulation levels of the *NbPOLA2* transcripts were calculated using the 2^−ΔΔ*C*t^ method (Livak and Schmittgen 2001). The expression level of the *NbActin* gene was detected and used as an internal control. For each treatment, four technical replicates were used and each treatment was repeated three times.

### 4.9 DNA Extraction and Viral Accumulation Level Analysis

The total DNA extraction and the absolute and relative quantification of TbCSV were conducted according to the method described previously (Sun et al. 2020; Li, Li et al. 2021; Mason et al. 2008). The quantification of viral and complementary strands in local infections was performed following a previous report (Rodríguez‐Negrete et al. 2014).

### 4.10 PVX‐Mediated Overexpression and Gene Silencing Assay

To overexpress the TbCSV *C3*, *C3* mutants and *NbPOLA2*, the coding sequences were inserted into the PVX vector and transformed into *A. tumefaciens* GV3101_(pSoup)_. The *Agrobacterium* cells were incubated for approximately 16 h at 28°C, and the cells were collected and resuspended using MMA buffer to OD_600_ = 1.0. Then the resuspended solution was inoculated into the leaves of *N. benthamiana* plants at the 4‐ to 6‐leaf stage using the agroinfiltration method.

To silence the *NbPOLA2* gene, a fragment of *NbPOLA2* with a size of about 500 bp was inserted into the PVX vector. The *Agrobacterium* transformation and inoculation were performed as described before.

The primers used in this study are listed in Table [Link], [Link].

## Conflicts of Interest

The authors declare no conflicts of interest.

## Supporting information


**FIGURE S1.** Detection of TbCSV *C3* gene expression in transgenic *Nicotiana benthamiana* plants. (a) Reverse transcription (RT)‐PCR detection of *C3* gene transcription in various transgenic *N. benthamiana* lines using *C3* gene‐specific primers. ‘L1 to L10’ indicate different transgenic *N. benthamiana* lines. ‘Nb’ indicates wild‐type *N. benthamiana* plants. RT‐PCR products were visualised by agarose gel electrophoresis. (b) RT‐quantitative PCR analyses of the relative transcription levels of *C3* in different transgenic lines. The transcription level of *C3* in line 1 was set as ‘1’. Statistical significance was determined by one‐way analysis of variance followed by Tukey’s multiple comparison test (*p* < 0.05), with different letters above each bar representing significant differences.

**FIGURE S2.** Reverse transcription (RT)‐PCR detection of TbCSV *C3* and *C3* mutant transcriptions expressed by the binary vector pCV (a) and the vector pGR106 (b). Expression vector’s promoter‐specific forward primer and gene‐specific reverse primers for *C3* and its mutants were used for PCR, and RT‐PCR products are visualised by agarose gel electrophoresis.

**FIGURE S3.** Relative accumulation of TbCSV_∆C3_ in *Nicotiana benthamiana* plants co‐infiltrated with pCV, pCV‐C3(1–122), pCV‐C3(1–129) or pCV‐C3. The leaf patches were harvested at 3 days post‐infiltration and subjected to quantitative PCR analyses. Four plants were analysed for each treatment and three technical replicates were used for each biological sample during quantitative PCR. Error bars represent means ± SEM. The different letters above each bar indicate statistically significant differences as determined by one‐way analysis of variance followed by Tukey’s multiple comparison test (*p* < 0.05). These experiments were performed with three independent biological replicates with similar results.

**FIGURE S4.** Subcellular localisation of TbCSV C3 and the truncated C3 mutant C3(1–122). C3 and C3 truncated mutant fused with green fluorescent protein (GFP) were transiently expressed in *Nicotiana benthamiana* leaf by agroinfiltration. Nuclear accumulations of C3 and C3 mutants are indicated by white arrows. Images were captured using laser scanning confocal microscopy at 3 days post agroinfiltration (dpai).

**FIGURE S5.** Yeast two‐hybrid (Y2H) analysis of intra‐ and inter‐molecular interactions of TYLCV C3. Recombinant plasmid pairs as that indicated were co‐transformed into yeast strain Y2H‐Gold. Transformants were streaked on SD/‐Trp‐Leu and SD/‐Trp‐Leu‐His‐Ade selective media. Co‐transformation with AD‐T+BK‐53 served as a positive control. Yeats co‐transformed with AD‐Lam+BK‐C3 was negative control. The *C3* and *C1* genes were cloned from TYLCV‐SH2 isolate (AM282874.1). Images were taken after 72 h.

**FIGURE S6.** Immunoblots for the expression of nYFP‐tagged proteins and cYFP‐tagged TbCSV C3. (a) Proteins used in the bimolecular fluorescence complementation (BiFC) assay in Figure 5 were detected with anti‐GFP‐N and anti‐GFP‐C antibodies respectively. For NbPCNA, a 6×His tag was fused and anti‐His antibody was used for detection. Coomassie brilliant blue (CBB)‐stained RuBisCO large subunit (RbcL) served as a loading control. (b) BiFC results of the interaction between TbCSV C3 and NbPCNA‐6×His.

**FIGURE S7.** Immunoblots for the expression of cYFP‐tagged TbCSV C3 and C3 mutants. Coomassie brilliant blue (CBB)‐stained RuBisCO large subunit (RbcL) served as a loading control.

**FIGURE S8.** Phenotypes induced by PVX‐mediated overexpression and silencing of *NbPOLA2* in *Nicotiana benthamiana* plants. (a) Symptoms induced by PVX‐NbPOLA2 or PVX‐Gus inoculations at 7 days post‐inoculation (dpi). (b) Relative accumulation of *NbPOLA2* in PVX‐NbPOLA2 and PVX‐GUS‐inoculated *N. benthamiana* plants, as determined by reverse transcription quantitative PCR (RT‐qPCR). (c) Symptoms induced by PVX‐Nbpola2 or PVX‐GUS inoculations at 7, 14 and 21 dpi. (d) PVX‐Nbpola2 inoculation‐mediated *NbPOLA2* silencing in *N. benthamiana* plants induced dwarf phenotype at 14 dpi. (e) Relative accumulation of *NbPOLA2* in PVX‐Nbpola2 and PVX‐GUS‐inoculated *N. benthamiana* plants. In (b) and (e), the upper new leaves were harvested at seven dpi for RT‐qPCR analysis. *Nicotiana benthamiana actin* was used as the internal reference. Four plants were analysed for each treatment and three technical replicates were used for each biological sample during qPCR. Error bars represent means ± SEM. Significant differences were determined by Student’s *t* test, **p < 0.01. These experiments were performed with three independent biological replicates with similar results.

**FIGURE S9.** Secondary structure formed in the 3′ end region of *C3* gene and *C3* mutants.

**FIGURE S10.** Protein sequence alignment of geminiviral C3/AC3 proteins. The abbreviated species names and their GenBank accession numbers are as follows: AbMV (Abutilon mosaic virus), LN611623.1; ACMV (African cassava mosaic virus), FN668378.1; AYVV (*Ageratum* yellow vein virus), LC487406.1; BCTV (beet curly top virus), MW182244.1; BGMV (bean golden mosaic virus), MT319763.1; CLCuMuV (cotton leaf curl Multan virus), KX656801.1; PYMV (potato yellow mosaic virus), NC_001934.1; TbCSV (tobacco curly shoot virus), AJ420318.1; TYLCYnV (tomato yellow leaf curl Yunnan virus), MN233597.1; ToLCV (tomato leaf curl virus), MH819291.1; ToLCKeV (tomato leaf curl Kerala virus), LN886521.1; TYLCCNV (tomato yellow leaf curl China virus), NC_004044.1; TYLCV (tomato yellow leaf curl virus), MK757243.1; MaYVV (*Malvastrum* yellow vein virus), NC_004634.1; TGMV (tomato golden mosaic virus), NC_001507.1; TLCYnV (tomato leaf curl Yunnan virus), HF674920.1.

**FIGURE S11.** Reverse transcription quantitative PCR analysis of the expression levels of *NbPOLA1.1/1.2*, *NbPOLA3.1/3.2*, *NbPOLA4.1* and *NbPOLA4.2* genes, with *NbActin* as the internal reference. Five plants were analysed for each treatment and three technical replicates were used for each biological sample. Data represent the mean of five independent biological replicates. Error bars represent means ± SEM. Significant differences were determined using Student’s *t* test: n.s., no significant difference, *p < 0.05, **p < 0.01. The experiment was repeated three times with similar results.

**TABLE S1.** Primers used in this study.

## Data Availability

The data that support the findings of this study are available from the corresponding author upon reasonable request.

## References

[mpp70068-bib-0001] Castillo, A. G. , D. Collinet , S. Deret , A. Kashoggi , and E. R. Bejarano . 2003. “Dual Interaction of Plant PCNA With Geminivirus Replication Accessory Protein (Ren) and Viral Replication Protein (Rep).” Virology 312: 381–394. 12919743 10.1016/s0042-6822(03)00234-4

[mpp70068-bib-0002] Chang, H. H. , C. H. Lee , C. J. Chang , and F. J. Jan . 2022. “FKBP‐Type Peptidyl‐Prolyl Cis‐Trans Isomerase Interacts With the Movement Protein of Tomato Leaf Curl New Delhi Virus and Impacts Viral Replication in *Nicotiana benthamiana* .” Molecular Plant Pathology 23: 561–575. 34984809 10.1111/mpp.13181 PMC8916215

[mpp70068-bib-0003] Chen, H. , Y. Zou , Y. Shang , et al. 2008. “Firefly Luciferase Complementation Imaging Assay for Protein–Protein Interactions in Plants.” Plant Physiology 146: 368–376. 18065554 10.1104/pp.107.111740 PMC2245818

[mpp70068-bib-0004] Elmer, J. S. , L. Brand , G. Sunter , W. E. Gardiner , D. M. Bisaro , and S. G. Rogers . 1988. “Genetic Analysis of the Tomato Golden Mosaic Virus. II. The Product of the AL1 Coding Sequence Is Required for Replication.” Nucleic Acids Research 16: 7043–7060. 3405758 10.1093/nar/16.14.7043 PMC338350

[mpp70068-bib-0005] Fondong, V. N. 2013. “Geminivirus Protein Structure and Function.” Molecular Plant Pathology 14: 635–649. 23615043 10.1111/mpp.12032 PMC6638828

[mpp70068-bib-0006] Fontes, E. P. , H. J. Gladfelter , R. L. Schaffer , I. T. Petty , and L. Hanley‐Bowdoin . 1994. “Geminivirus Replication Origins Have a Modular Organization.” Plant Cell 6: 405–416. 8180499 10.1105/tpc.6.3.405 PMC160443

[mpp70068-bib-0052] Gong, P. , H. Tan , S. Zhao , et al. 2021. “Geminiviruses Encode Additional Small Proteins with Specific Subcellular Localizations and Virulence Function.” Nature Communications 12: 4278. 10.1038/s41467-021-24617-4 PMC8277811 34257307

[mpp70068-bib-0007] Gong, P. , S. Zhao , H. Liu , Z. Chang , F. Li , and X. Zhou . 2022. “Tomato Yellow Leaf Curl Virus V3 Protein Traffics Along Microfilaments to Plasmodesmata to Promote Virus Cell‐To‐Cell Movement.” Science China. Life Sciences 65: 1046–1049. 35226256 10.1007/s11427-021-2063-4

[mpp70068-bib-0008] Hanley‐Bowdoin, L. , E. R. Bejarano , D. Robertson , and S. Mansoor . 2013. “Geminiviruses: Masters at Redirecting and Reprogramming Plant Processes.” Nature Reviews Microbiology 11: 777–788. 24100361 10.1038/nrmicro3117

[mpp70068-bib-0009] Hanley‐Bowdoin, L. , S. B. Settlage , B. M. Orozco , S. Nagar , and D. Robertson . 2000. “Geminiviruses: Models for Plant DNA Replication, Transcription, and Cell Cycle Regulation.” Critical Reviews in Biochemistry and Molecular Biology 35: 105–140. 10821479

[mpp70068-bib-0010] Hefferon, K. L. , Y. S. Moon , and Y. Fan . 2006. “Multi‐Tasking of Nonstructural Gene Products Is Required for Bean Yellow Dwarf Geminivirus Transcriptional Regulation.” FEBS Journal 273: 4482–4494. 16972938 10.1111/j.1742-4658.2006.05454.x

[mpp70068-bib-0011] Jain, R. , A. K. Aggarwal , and O. Rechkoblit . 2018. “Eukaryotic DNA Polymerases.” Current Opinion in Structural Biology 53: 77–87. 30005324 10.1016/j.sbi.2018.06.003

[mpp70068-bib-0012] Jeske, H. , M. Lütgemeier , and W. Preiss . 2001. “DNA Forms Indicate Rolling Circle and Recombination‐Dependent Replication of Abutilon Mosaic Virus.” EMBO Journal 20: 6158–6167. 11689455 10.1093/emboj/20.21.6158 PMC125697

[mpp70068-bib-0013] Li, F. , R. Qiao , Z. Wang , X. Yang , and X. Zhou . 2022. “Occurrence and Distribution of Geminiviruses in China.” Science China. Life Sciences 65: 1498–1503. 35661965 10.1007/s11427-022-2125-2

[mpp70068-bib-0014] Li, F. , X. Xu , C. Huang , et al. 2015. “The AC5 Protein Encoded by *Mungbean yellow mosaic india virus* Is a Pathogenicity Determinant That Suppresses RNA Silencing‐Based Antiviral Defenses.” New Phytologist 208: 555–569. 26010321 10.1111/nph.13473

[mpp70068-bib-0015] Li, M. , C. Li , K. Jiang , et al. 2021. “Characterization of Pathogenicity‐Associated V2 Protein of Tobacco Curly Shoot Virus.” International Journal of Molecular Sciences 22: 923. 33477652 10.3390/ijms22020923 PMC7831499

[mpp70068-bib-0016] Li, M. , J. Zhang , M. Feng , et al. 2018. “Characterization of Silencing Suppressor p24 of *Grapevine leafroll‐associated virus 2* .” Molecular Plant Pathology 19: 355–368. 27997767 10.1111/mpp.12525 PMC6638178

[mpp70068-bib-0017] Li, P. , L. Guo , X. Lang , et al. 2022. “Geminivirus C4 Proteins Inhibit GA Signaling via Prevention of NbGAI Degradation, to Promote Viral Infection and Symptom Development in *N. benthamiana* .” PLoS Pathogens 18: e1010217. 35390110 10.1371/journal.ppat.1010217 PMC9060335

[mpp70068-bib-0018] Li, P. , F. Su , Q. Meng , et al. 2021. “The C5 Protein Encoded by Ageratum Leaf Curl Sichuan Virus Is a Virulence Factor and Contributes to the Virus Infection.” Molecular Plant Pathology 22: 1149–1158. 34219358 10.1111/mpp.13103 PMC8359000

[mpp70068-bib-0019] Li, Z. , G. Li , Y. Xie , Z. Zhang , and X. Zhou . 2002. “Tomato Leaf Curl Disease in Yunnan Is Caused by Tobacco Curly Shoot Virus.” Chinese Journal of Virology 18: 355–361.

[mpp70068-bib-0020] Li, Z. , Y. Xie , and X. Zhou . 2005. “Tobacco Curly Shoot Virus DNAβ Is Not Necessary for Infection but Intensifies Symptoms in a Host‐Dependent Manner.” Phytopathology 95: 902–908. 18944412 10.1094/PHYTO-95-0902

[mpp70068-bib-0021] Liu, H. , Z. Chang , S. Zhao , et al. 2023. “Functional Identification of a Novel C7 Protein of Tomato Yellow Leaf Curl Virus.” Virology 585: 117–126. 37331112 10.1016/j.virol.2023.05.011

[mpp70068-bib-0022] Livak, K. J. , and T. D. Schmittgen . 2001. “Analysis of Relative Gene Expression Data Using Real‐Time Quantitative PCR and the 2^−∆∆Ct^ Method.” Methods 25: 402–408. 11846609 10.1006/meth.2001.1262

[mpp70068-bib-0023] Mason, G. , P. Caciagli , G. P. Accotto , and E. Noris . 2008. “Real‐Time PCR for the Quantitation of *Tomato yellow leaf curl Sardinia virus* in Tomato Plants and in *Bemisia tabaci* .” Journal of Virological Methods 147: 282–289. 17980920 10.1016/j.jviromet.2007.09.015

[mpp70068-bib-0024] Morris, B. , K. Richardson , P. Eddy , X. C. Zhan , A. Haley , and R. Gardner . 1991. “Mutagenesis of the AC3 Open Reading Frame of *African cassava mosaic virus* DNA A Reduces DNA B Replication and Ameliorates Disease Symptoms.” Journal of General Virology 72: 1205–1213. 2045787 10.1099/0022-1317-72-6-1205

[mpp70068-bib-0025] Muhire, B. M. , M. Golden , B. Murrell , et al. 2014. “Evidence of Pervasive Biologically Functional Secondary Structures Within the Genomes of Eukaryotic Single‐Stranded DNA Viruses.” Journal of Virology 88: 1972–1989. 24284329 10.1128/JVI.03031-13 PMC3911531

[mpp70068-bib-0026] Orozco, B. M. , H. J. Gladfelter , S. B. Settlage , P. A. Eagle , R. N. Gentry , and L. Hanley‐Bowdoin . 1998. “Multiple Cis Elements Contribute to Geminivirus Origin Function.” Virology 242: 346–356. 9514968 10.1006/viro.1997.9013

[mpp70068-bib-0027] Orozco, B. M. , and L. Hanley‐Bowdoin . 1996. “A DNA Structure Is Required for Geminivirus Replication Origin Function.” Journal of Virology 70: 148–158. 8523519 10.1128/jvi.70.1.148-158.1996 PMC189799

[mpp70068-bib-0028] Pasumarthy, K. K. , N. R. Choudhury , and S. K. Mukherjee . 2010. “ *Tomato leaf curl Kerala virus* (ToLCKeV) AC3 Protein Forms a Higher Order Oligomer and Enhances ATPase Activity of Replication Initiator Protein (Rep/AC1).” Virology Journal 7: 128. 20546567 10.1186/1743-422X-7-128 PMC2901266

[mpp70068-bib-0029] Pasumarthy, K. K. , S. K. Mukherjee , and N. R. Choudhury . 2011. “The Presence of Tomato Leaf Curl Kerala Virus AC3 Protein Enhances Viral DNA Replication and Modulates Virus Induced Gene‐Silencing Mechanism in Tomato Plants.” Virology Journal 8: 178. 21496351 10.1186/1743-422X-8-178 PMC3102638

[mpp70068-bib-0030] Rizvi, I. , N. R. Choudhury , and N. Tuteja . 2015. “Insights Into the Functional Characteristics of Geminivirus Rolling‐Circle Replication Initiator Protein and Its Interaction With Host Factors Affecting Viral DNA Replication.” Archives of Virology 160: 375–387. 25449306 10.1007/s00705-014-2297-7

[mpp70068-bib-0031] Rodríguez‐Negrete, E. A. , S. Sánchez‐Campos , M. C. Cañizares , et al. 2014. “A Sensitive Method for the Quantification of Virion‐Sense and Complementary‐Sense DNA Strands of Circular Single‐Stranded DNA Viruses.” Scientific Reports 4: 6438. 25241765 10.1038/srep06438 PMC5377365

[mpp70068-bib-0032] Rojas, M. R. , C. Hagen , W. J. Lucas , and R. L. Gilbertson . 2005. “Exploiting Chinks in the Plant's Armor: Evolution and Emergence of Geminiviruses.” Annual Review of Phytopathology 43: 361–394. 10.1146/annurev.phyto.43.040204.135939 16078889

[mpp70068-bib-0033] Selth, L. A. , S. C. Dogra , M. S. Rasheed , H. Healy , J. W. Randles , and M. A. Rezaian . 2005. “A NAC Domain Protein Interacts With Tomato Leaf Curl Virus Replication Accessory Protein and Enhances Viral Replication.” Plant Cell 17: 311–325. 15608335 10.1105/tpc.104.027235 PMC544507

[mpp70068-bib-0034] Settlage, S. B. , A. B. Miller , W. Gruissem , and L. Hanley‐Bowdoin . 2001. “Dual Interaction of a Geminivirus Replication Accessory Factor With a Viral Replication Protein and a Plant Cell Cycle Regulator.” Virology 279: 570–576. 11162812 10.1006/viro.2000.0719

[mpp70068-bib-0035] Settlage, S. B. , A. B. Miller , and L. Hanley‐Bowdoin . 1996. “Interactions Between Geminivirus Replication Proteins.” Journal of Virology 70: 6790–6795. 8794317 10.1128/jvi.70.10.6790-6795.1996 PMC190723

[mpp70068-bib-0036] Settlage, S. B. , R. G. See , and L. Hanley‐Bowdoin . 2005. “Geminivirus C3 Protein: Replication Enhancement and Protein Interactions.” Journal of Virology 79: 9885–9895. 16014949 10.1128/JVI.79.15.9885-9895.2005 PMC1181577

[mpp70068-bib-0037] Shen, R. , and W. A. Miller . 2004. “The 3′ Untranslated Region of Tobacco Necrosis Virus RNA Contains a Barley Yellow Dwarf Virus‐Like Cap‐Independent Translation Element.” Journal of Virology 78: 4655–4664. 15078948 10.1128/JVI.78.9.4655-4664.2004 PMC387721

[mpp70068-bib-0038] Stockley, P. G. , R. Twarock , S. E. Bakker , et al. 2013. “Packaging Signals in Single‐Stranded RNA Viruses: Nature's Alternative to a Purely Electrostatic Assembly Mechanism.” Journal of Biological Physics 39: 277–287. 23704797 10.1007/s10867-013-9313-0 PMC3662417

[mpp70068-bib-0039] Sun, M. , K. Jiang , C. Li , et al. 2020. “ *Tobacco curly shoot virus* C3 Protein Enhances Viral Replication and Gene Expression in *Nicotiana benthamiana* Plants.” Virus Research 281: 197939. 32198077 10.1016/j.virusres.2020.197939

[mpp70068-bib-0040] Sung, Y. K. , and R. H. Coutts . 1995a. “Mutational Analysis of Potato Yellow Mosaic Geminivirus.” Journal of General Virology 76: 1773–1780. 9049382 10.1099/0022-1317-76-7-1773

[mpp70068-bib-0041] Sung, Y. K. , and R. H. Coutts . 1995b. “Pseudorecombination and Complementation Between Potato Yellow Mosaic Geminivirus and Tomato Golden Mosaic Geminivirus.” Journal of General Virology 76: 2809–2815. 7595388 10.1099/0022-1317-76-11-2809

[mpp70068-bib-0042] Sunter, G. , M. D. Hartitz , S. G. Hormuzdi , C. L. Brough , and D. M. Bisaro . 1990. “Genetic Analysis of Tomato Golden Mosaic Virus: ORF AL2 Is Required for Coat Protein Accumulation While ORF AL3 Is Necessary for Efficient DNA Replication.” Virology 179: 69–77. 2219741 10.1016/0042-6822(90)90275-v

[mpp70068-bib-0043] Wang, D. , C. Yang , Y. Deng , et al. 2022. “Conserved RNA Secondary Structure in Cherry Virus A 5′‐UTR Associated With Translation Regulation.” Virology Journal 19: 91. 35619168 10.1186/s12985-022-01824-z PMC9137147

[mpp70068-bib-0044] Wang, Z. , Y. Wang , R. Lozano‐Duran , T. Hu , and X. Zhou . 2022. “Identification of a Novel C6 Protein Encoded by Tomato Leaf Curl China Virus.” Phytopathology Research 4: 46.

[mpp70068-bib-0045] Wikström, F. H. , B. M. Meehan , M. Berg , et al. 2007. “Structure‐Dependent Modulation of α‐Interferon Production by Porcine Circovirus 2 Oligodeoxyribonucleotide and CpG DNAs in Porcine Peripheral Blood Mononuclear Cells.” Journal of Virology 81: 4919–4927. 17329341 10.1128/JVI.02797-06 PMC1900218

[mpp70068-bib-0046] Wu, M. , X. Ding , X. Fu , and R. Lozano‐Duran . 2019. “Transcriptional Reprogramming Caused by the Geminivirus Tomato Yellow Leaf Curl Virus in Local or Systemic Infections in *Nicotiana benthamiana* .” BMC Genomics 20: 542. 31272383 10.1186/s12864-019-5842-7 PMC6611054

[mpp70068-bib-0047] Wu, M. , H. Wei , H. Tan , et al. 2021. “Plant DNA Polymerases α and δ Mediate Replication of Geminiviruses.” Nature Communications 12: 2780. 10.1038/s41467-021-23013-2 PMC8119979 33986276

[mpp70068-bib-0048] Xie, Y. , X. Zhou , Z. Zhang , and Y. Qi . 2002. “Tobacco Curly Shoot Virus Isolated in Yunnan Is a Distinct Species of *Begomovirus* .” Chinese Science Bulletin 47: 199–201.

[mpp70068-bib-0049] Yan, Z. Y. , L. Fang , X. J. Xu , et al. 2022. “A Predicted Stem Loop in Coat Protein‐Coding Sequence of Tobacco Vein Banding Mosaic Virus Is Required for Efficient Replication.” Phytopathology 112: 441–451. 34191551 10.1094/PHYTO-10-20-0463-R

[mpp70068-bib-0050] Yang, X. , W. Guo , F. Li , G. Sunter , and X. Zhou . 2019. “Geminivirus‐Associated Betasatellites: Exploiting Chinks in the Antiviral Arsenal of Plants.” Trends in Plant Science 24: 519–529. 31003895 10.1016/j.tplants.2019.03.010

[mpp70068-bib-0051] Zhou, X. 2013. “Advances in Understanding Begomovirus Satellites.” Annual Review of Phytopathology 51: 357–381. 10.1146/annurev-phyto-082712-102234 23915133

