# Supplementary figures and images for: The Role of a C‐Terminal Seven‐Amino Acid Motif in TbCSV C3 Protein and Its Interaction With NbPOLA2 in Enhancing Viral Replication
Source: Mol Plant Pathol. 2025 Mar 2;26(3):e70068. doi: 10.1111/mpp.70068 (PMC11872800; doi:10.1111/mpp.70068)

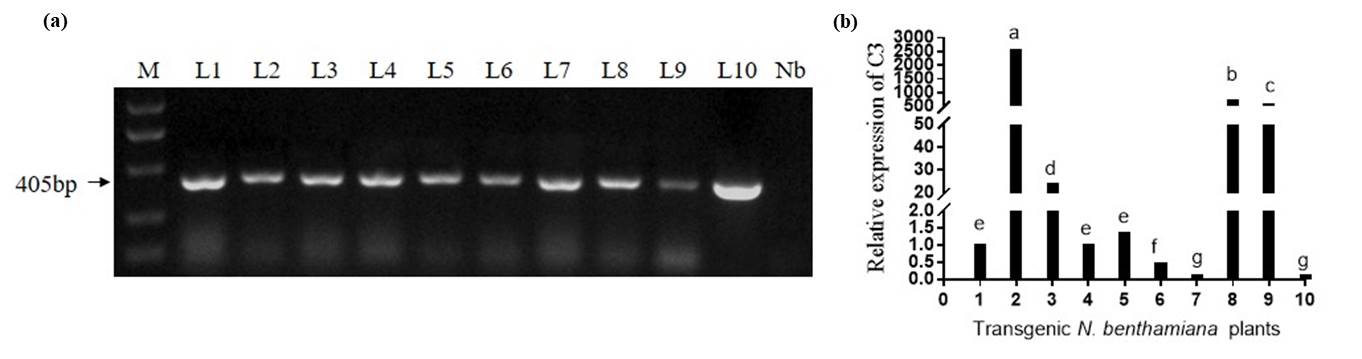

Supplement: Supplementary file 1 — FIGURE S1. Detection of TbCSV C3 gene expression in transgenic Nicotiana benthamiana plants. (a) Reverse transcription (RT)‐PCR detection of C3 gene transcription in various transgenic N. benthamiana lines using C3 gene‐specific primers. ‘L1 to L10’ indicate different transgenic N. benthamiana lines. ‘Nb’ indicates wild‐type N. benthamiana plants. RT‐PCR products were visualised by agarose gel electrophoresis. (b) RT‐quantitative PCR analyses of the relative transcription levels of C3 in different transgenic lines. The transcription level of C3 in line 1 was set as ‘1’. Statistical significance was determined by one‐way analysis of variance followed by Tukey’s multiple comparison test (p < 0.05), with different letters above each bar representing significant differences. [file MPP-26-e70068-s011.jpg]

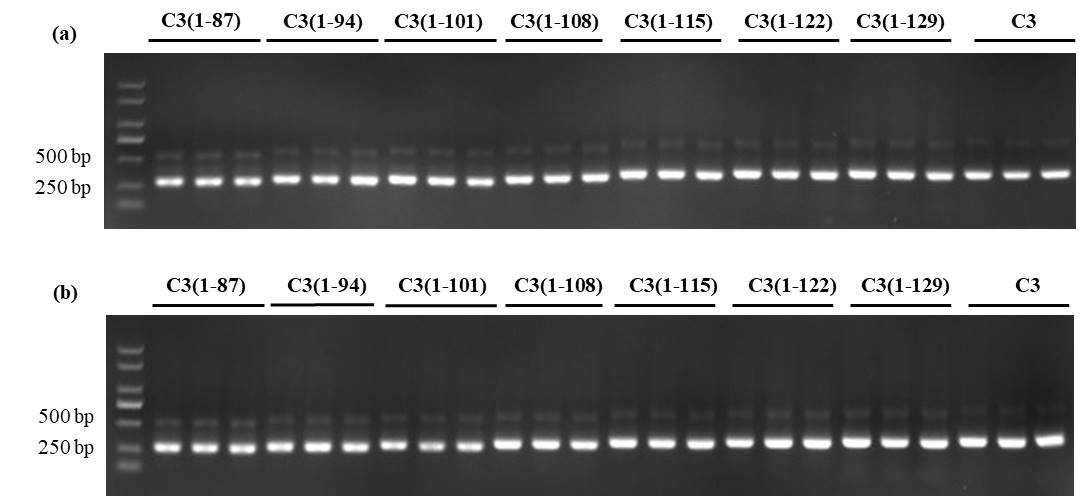

Supplement: Supplementary file 2 — FIGURE S2. Reverse transcription (RT)‐PCR detection of TbCSV C3 and C3 mutant transcriptions expressed by the binary vector pCV (a) and the vector pGR106 (b). Expression vector’s promoter‐specific forward primer and gene‐specific reverse primers for C3 and its mutants were used for PCR, and RT‐PCR products are visualised by agarose gel electrophoresis. [file MPP-26-e70068-s002.jpg]

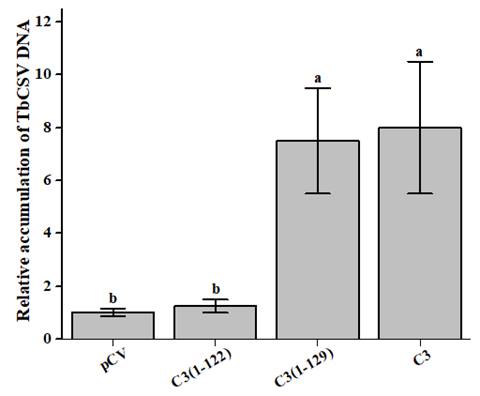

Supplement: Supplementary file 3 — FIGURE S3. Relative accumulation of TbCSV∆C3 in Nicotiana benthamiana plants co‐infiltrated with pCV, pCV‐C3(1–122), pCV‐C3(1–129) or pCV‐C3. The leaf patches were harvested at 3 days post‐infiltration and subjected to quantitative PCR analyses. Four plants were analysed for each treatment and three technical replicates were used for each biological sample during quantitative PCR. Error bars represent means ± SEM. The different letters above each bar indicate statistically significant differences as determined by one‐way analysis of variance followed by Tukey’s multiple comparison test (p < 0.05). These experiments were performed with three independent biological replicates with similar results. [file MPP-26-e70068-s007.jpg]

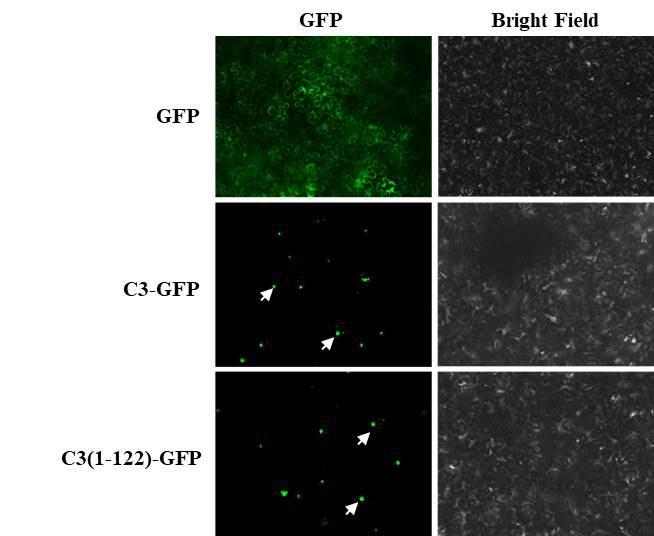

Supplement: Supplementary file 4 — FIGURE S4. Subcellular localisation of TbCSV C3 and the truncated C3 mutant C3(1–122). C3 and C3 truncated mutant fused with green fluorescent protein (GFP) were transiently expressed in Nicotiana benthamiana leaf by agroinfiltration. Nuclear accumulations of C3 and C3 mutants are indicated by white arrows. Images were captured using laser scanning confocal microscopy at 3 days post agroinfiltration (dpai). [file MPP-26-e70068-s009.jpg]

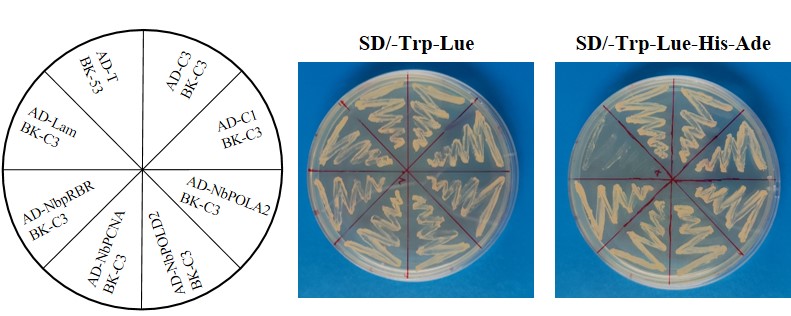

Supplement: Supplementary file 5 — FIGURE S5. Yeast two‐hybrid (Y2H) analysis of intra‐ and inter‐molecular interactions of TYLCV C3. Recombinant plasmid pairs as that indicated were co‐transformed into yeast strain Y2H‐Gold. Transformants were streaked on SD/‐Trp‐Leu and SD/‐Trp‐Leu‐His‐Ade selective media. Co‐transformation with AD‐T+BK‐53 served as a positive control. Yeats co‐transformed with AD‐Lam+BK‐C3 was negative control. The C3 and C1 genes were cloned from TYLCV‐SH2 isolate (AM282874.1). Images were taken after 72 h. [file MPP-26-e70068-s006.jpg]

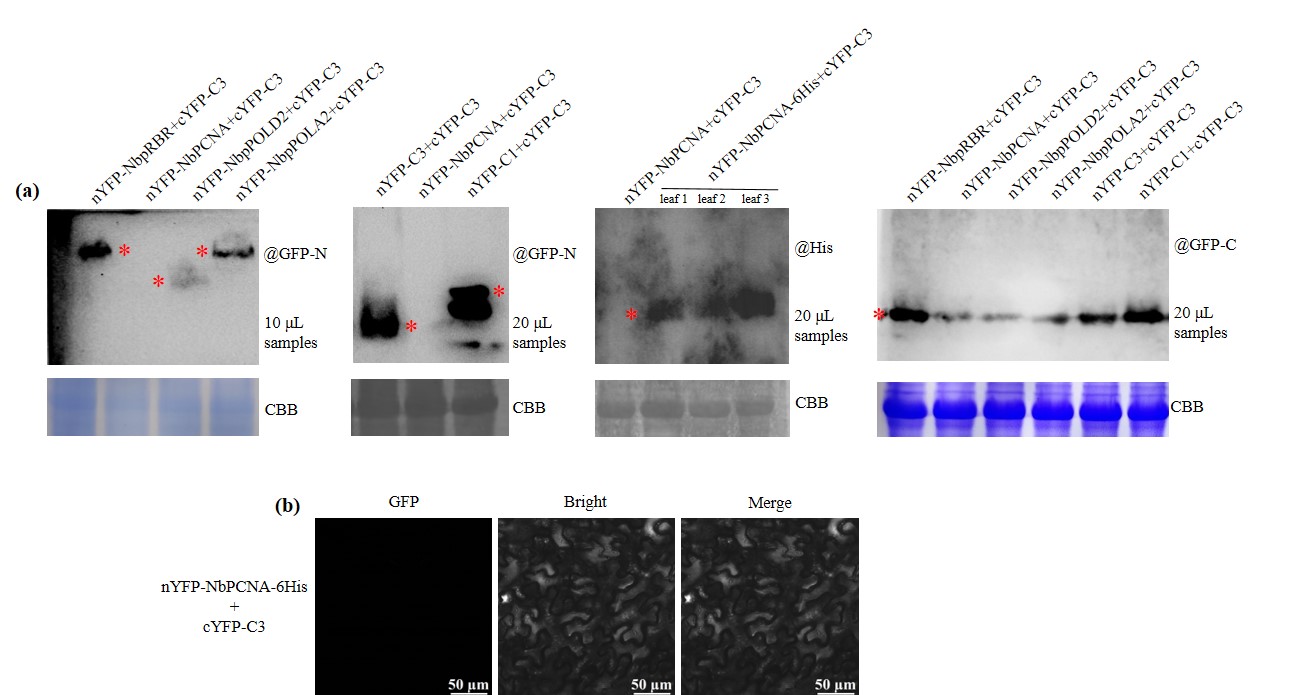

Supplement: Supplementary file 6 — FIGURE S6. Immunoblots for the expression of nYFP‐tagged proteins and cYFP‐tagged TbCSV C3. (a) Proteins used in the bimolecular fluorescence complementation (BiFC) assay in Figure 5 were detected with anti‐GFP‐N and anti‐GFP‐C antibodies respectively. For NbPCNA, a 6×His tag was fused and anti‐His antibody was used for detection. Coomassie brilliant blue (CBB)‐stained RuBisCO large subunit (RbcL) served as a loading control. (b) BiFC results of the interaction between TbCSV C3 and NbPCNA‐6×His. [file MPP-26-e70068-s004.jpg]

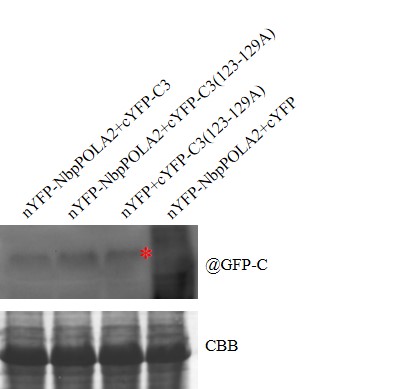

Supplement: Supplementary file 7 — FIGURE S7. Immunoblots for the expression of cYFP‐tagged TbCSV C3 and C3 mutants. Coomassie brilliant blue (CBB)‐stained RuBisCO large subunit (RbcL) served as a loading control. [file MPP-26-e70068-s012.jpg]

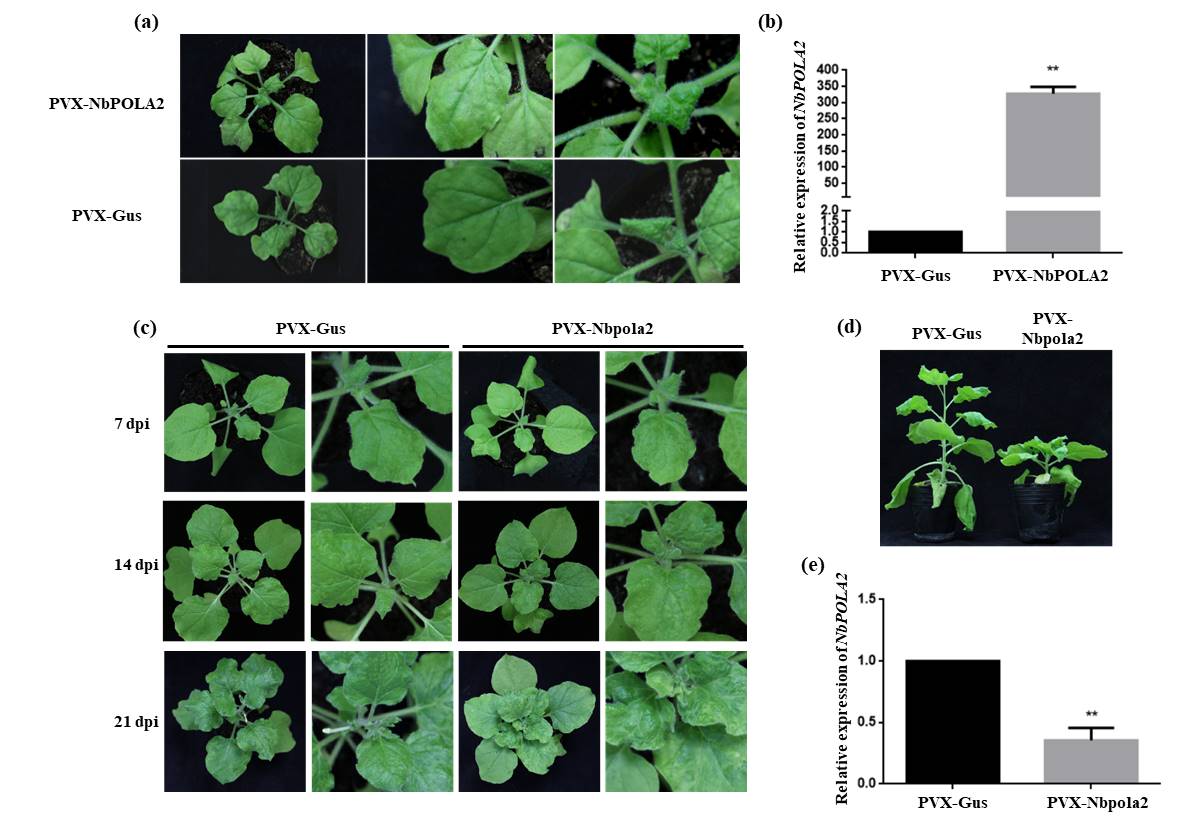

Supplement: Supplementary file 8 — FIGURE S8. Phenotypes induced by PVX‐mediated overexpression and silencing of NbPOLA2 in Nicotiana benthamiana plants. (a) Symptoms induced by PVX‐NbPOLA2 or PVX‐Gus inoculations at 7 days post‐inoculation (dpi). (b) Relative accumulation of NbPOLA2 in PVX‐NbPOLA2 and PVX‐GUS‐inoculated N. benthamiana plants, as determined by reverse transcription quantitative PCR (RT‐qPCR). (c) Symptoms induced by PVX‐Nbpola2 or PVX‐GUS inoculations at 7, 14 and 21 dpi. (d) PVX‐Nbpola2 inoculation‐mediated NbPOLA2 silencing in N. benthamiana plants induced dwarf phenotype at 14 dpi. (e) Relative accumulation of NbPOLA2 in PVX‐Nbpola2 and PVX‐GUS‐inoculated N. benthamiana plants. In (b) and (e), the upper new leaves were harvested at seven dpi for RT‐qPCR analysis. Nicotiana benthamiana actin was used as the internal reference. Four plants were analysed for each treatment and three technical replicates were used for each biological sample during qPCR. Error bars represent means ± SEM. Significant differences were determined by Student’s t test, **p < 0.01. These experiments were performed with three independent biological replicates with similar results. [file MPP-26-e70068-s008.jpg]

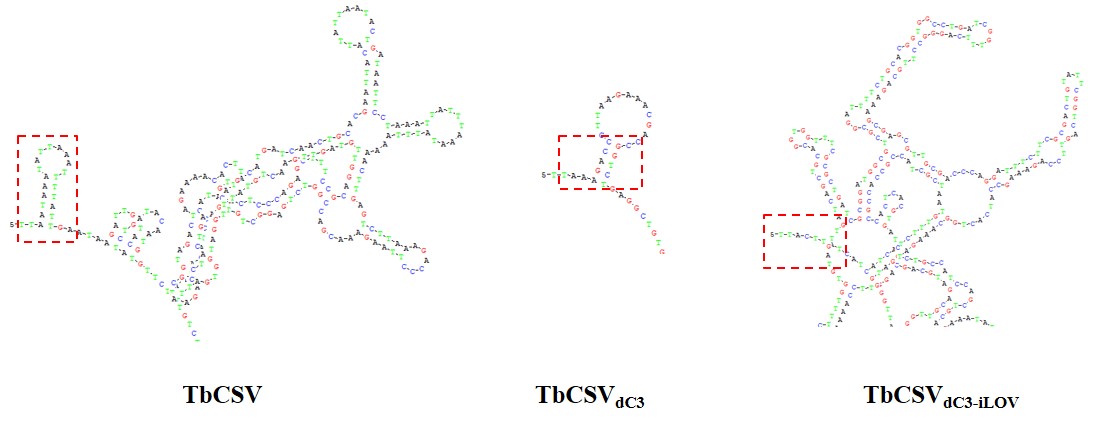

Supplement: Supplementary file 9 — FIGURE S9. Secondary structure formed in the 3′ end region of C3 gene and C3 mutants. [file MPP-26-e70068-s001.jpg]

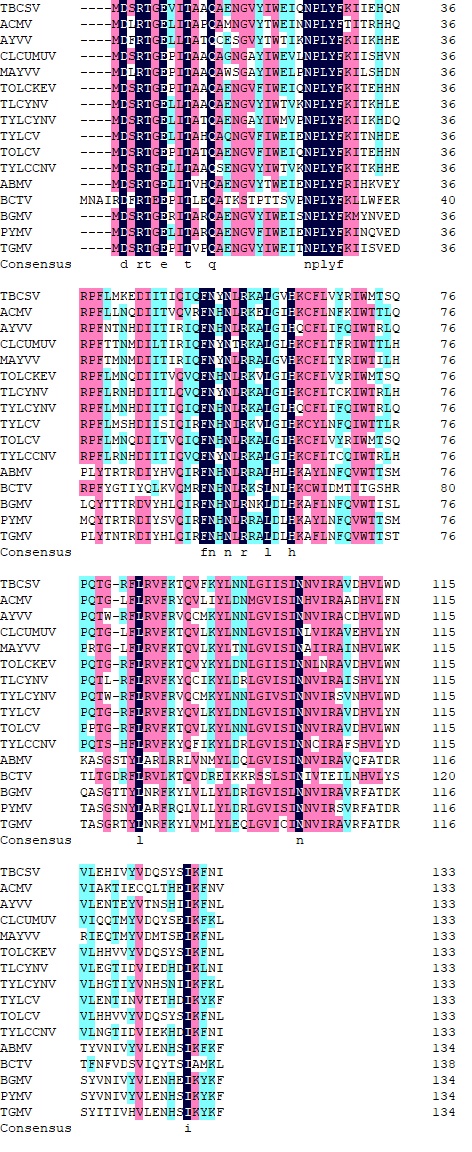

Supplement: Supplementary file 10 — FIGURE S10. Protein sequence alignment of geminiviral C3/AC3 proteins. The abbreviated species names and their GenBank accession numbers are as follows: AbMV (Abutilon mosaic virus), LN611623.1; ACMV (African cassava mosaic virus), FN668378.1; AYVV (Ageratum yellow vein virus), LC487406.1; BCTV (beet curly top virus), MW182244.1; BGMV (bean golden mosaic virus), MT319763.1; CLCuMuV (cotton leaf curl Multan virus), KX656801.1; PYMV (potato yellow mosaic virus), NC_001934.1; TbCSV (tobacco curly shoot virus), AJ420318.1; TYLCYnV (tomato yellow leaf curl Yunnan virus), MN233597.1; ToLCV (tomato leaf curl virus), MH819291.1; ToLCKeV (tomato leaf curl Kerala virus), LN886521.1; TYLCCNV (tomato yellow leaf curl China virus), NC_004044.1; TYLCV (tomato yellow leaf curl virus), MK757243.1; MaYVV (Malvastrum yellow vein virus), NC_004634.1; TGMV (tomato golden mosaic virus), NC_001507.1; TLCYnV (tomato leaf curl Yunnan virus), HF674920.1. [file MPP-26-e70068-s003.jpg]

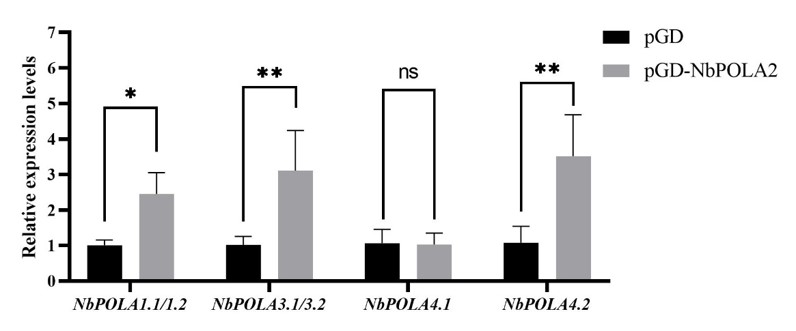

Supplement: Supplementary file 11 — FIGURE S11. Reverse transcription quantitative PCR analysis of the expression levels of NbPOLA1.1/1.2, NbPOLA3.1/3.2, NbPOLA4.1 and NbPOLA4.2 genes, with NbActin as the internal reference. Five plants were analysed for each treatment and three technical replicates were used for each biological sample. Data represent the mean of five independent biological replicates. Error bars represent means ± SEM. Significant differences were determined using Student’s t test: n.s., no significant difference, *p < 0.05, **p < 0.01. The experiment was repeated three times with similar results. [file MPP-26-e70068-s005.jpg]
