# Supplementary material for: The Role of a C‐Terminal Seven‐Amino Acid Motif in TbCSV C3 Protein and Its Interaction With NbPOLA2 in Enhancing Viral Replication
Source: Mol Plant Pathol. 2025 Mar 2;26(3):e70068. doi: 10.1111/mpp.70068 (PMC11872800; doi:10.1111/mpp.70068)
Supplement: Supplementary file 12 — TABLE S1. Primers used in this study. [file MPP-26-e70068-s010.docx]

**Table S1. Cloning and quantitative PCR primers used in this study**

| Primer name | Primer sequence (5’-3’) |
| --- | --- |
| Y35bF | GGATCCATTAGTAAACGAGTTTC |
| Y35bR | GGATCCCACATAGTGCGGAGTGC |
| dC3-F | CTATGATTCCGTAACAAATTACTTAAAGACCCTTAAGA |
| dC3-R | TCTTAAGGGTCTTTAAGTAATTTGTTACGGAATCATAG |
| pCV-C3-F | GGATCCATGGATTCACGCACAGGGG |
| pCV-C3-R | GAGCTCTTAATAAATATTAAATTTTATTG |
| pCV-C3(1-87)-R | GAGCTCTTACTTAAAGACCCTTAAGAAACG |
| pCV-C3(1-94)-R | GAGCTCTTATAAATATTTAAAAACTTGAGT |
| pCV-C3(1-101)-R | GAGCTCTTAACTGATAATTCCTAAATTATT |
| pCV-C3(1-108)-R | GAGCTCTTATGCACGAATTACATTATTAAT |
| pCV-C3(1-115)-R | GAGCTCTTAATCCCATAATACATGATCAAC |
| pCV-C3(1-122)-R | GAGCTCTTAATATACAATGTGTTCTAATAC |
| pCV-C3(1-129)-R | GAGCTCTTATATTGAATAAGATTGGTCTAC |
| PVX-C3-F | CCATCGATATGGATTCACGCACAGGGG |
| PVX-C3-R | GCATCGATTTAATAAATATTAAATTTTATTGA |
| PVX-C3(1-87)-R | GCATCGATTTACTTAAAGACCCTTAAGAAACG |
| PVX-C3(1-94)-R | GCATCGATTTATAAATATTTAAAAACTTGAGT |
| PVX-C3(1-101)-R | GCATCGATTTAACTGATAATTCCTAAATTATT |
| PVX-C3(1-108)-R | GCATCGATTTATGCACGAATTACATTATTAAT |
| PVX-C3(1-115)-R | GCATCGATTTAATCCCATAATACATGATCAAC |
| PVX-C3(1-122)-R | GCATCGATTTAATATACAATGTGTTCTAATAC |
| PVX-C3(1-129)-R | GCATCGATTTATATTGAATAAGATTGGTCTAC |
| Y35-C3(d123-129)-F | TATTAAATTTATATACAATGTGTTCTAATACATCCCA |
| Y35-C3(d123-129)-R | CATTGTATATAAATTTAATATTTATTAATTTGTTAC |
| 1.9-C-F | AATTGTTTTGTGGTCCCTTATTTA |
| 1.9-C-R | ACATGTTAAAAACCTCTCCAAAA |
| BK-C3-F | TGCATATGGCCATGGAGGCCGAATTCATGGATTCACGCACAGGG |
| BK-C3-R | TGCGGCCGCTGCAGGTCGACGGATCCTTAATAAATATTAAATTTTA |
| AD-C3-F | TATGGCCATGGAGGCCAGTGAATTCATGGATTCACGCACAGGG |
| AD-C3-R | TCTGCAGCTCGAGCTCGATGGATCCTTAATAAATATTAAATTTTA |
| AD-C1-F | TATGGCCATGGAGGCCAGTGAATTCATGCCTCAGCCAAGAAAA |
| AD-C1-R | TCTGCAGCTCGAGCTCGATGGATCCTCAACACGACGACGTCTG |
| AD-NbPOLA2-F | ATATGGCCATGGAGGCCAGTGAATTCATGGAAGAGCAAATCAAAGCT |
| AD-NbPOLA2-R | ATCTGCAGCTCGAGCTCGATGGATCCCTATATACGAATAACTGAAGC |
| AD-NbPOLD2-F | TATGGCCATGGAGGCCAGTGAATTCATGGTCGTAGCAATGG |
| AD-NbPOLD2-R | TCTGCAGCTCGAGCTCGATGGATCCTTATGAGTGGATTTGAG |
| AD-NbPCNA-F | TATGGCCATGGAGGCCAGTGAATTCATGTTGGAATTACGGCTTG |
| AD-NbPCNA-R | TCTGCAGCTCGAGCTCGATGGATCCTCAAGGTTTGGTTTCCTCT |
| AD-NbpRBR-F | ATGGCCATGGAGGCCAGTGAATTCATGGTGGAGCTGAATAATTGTTC |
| AD-NbpRBR-R | CTGCAGCTCGAGCTCGATGGATCCCTAAGACTCAGGCTGCTCAGTTT |
| BK-C3(1-129)-R | TGCGGCCGCTGCAGGTCGACGGATCCTTATATTGAATAAGATTGGTC |
| BK-C3(85-134)-F | TGCATATGGCCATGGAGGCCGAATTCATGTTTAAGACTCAAGTT |
| BK-C3(1-99)-R | TGCGGCCGCTGCAGGTCGACGGATCCTTAAATTCCTAAATTATTT |
| BK-C3(1-119)-R | TGCGGCCGCTGCAGGTCGACGGATCCTTAGTGTTCTAATACATC |
| BK-C3(1-123)-F | aaGAATTCATGGATTCACGCACAGGG |
| BK-C3(1-123)-R | cgGGATCCCTATACATATACAATGTGTTC |
| BK-C3(55-119)-F | TGCATATGGCCATGGAGGCCGAATTCATGCTGCGGAAAGCTCTGGGA |
| BK-C3(61-119)-F | TGCATATGGCCATGGAGGCCGAATTCATGGGAGTGCACAAGTGTTTT |
| pCV-nYFP-POLD2-F | AACATCGAGGACTCCGGAGTCGACATGGTCGTAGCAATGG |
| pCV-nYFP-POLD2-R | GAATTCGAGCTCGCCTGGGGATCCTTATGAGTGGATTTGAG |
| pCV-cYFP-C3-F | GAGCTGTACAAGTCCGGAGTCGACATGGATTCACGCACAGGG |
| pCV-cYFP-C3-R | GAATTCGAGCTCGCCTGGGGATCCTTAATAAATATTAAATTTTA |
| pCV-nYFP-POLA2-F | AACATCGAGGACTCCGGAGTCGACATGGAAGAGCAAATCA |
| pCV-nYFP-POLA2-R | GAATTCGAGCTCGCCTGGGGATCCCTATATACGAATAACTG |
| cLUC-POLA2-F | TACGCGTCCCGGGGCGGTACCATGACAATTAAATACAACAACA |
| cLUC-POLA2-R | ACGAAAGCTCTGCAGGTCGACTCATATACGAATAACTGAAGC |
| nLUC-C3-F | GGGGACGAGCTCGGTACCATGGATTCACGCACAGGG |
| nLUC-C3-R | CGCGTACGAGATCTGGTCGACATAAATATTAAATTTTA |
| PVX-NbPOLA2-F | GTCAGCACCAGCTAGCATCGATATGGAAGAGCAAATCAAAGCTGA |
| PVX-NbPOLA2-R | AACCGTTCATCGGCGGTCGACCTATATACGAATAACTGAAGC |
| PVX-Nbpola2-F | GTCAGCACCAGCTAGCATCGATAGGAGAACCCCAATGATG |
| PVX-Nbpola2-R | AACCGTTCATCGGCGGTCGACCGCTGCTTTGCAGTAAAA |
| pCV-NbPOLA2 -F | TGGAGAGAACACGGGGGACTCTAGAATGGAAGAGCAAATCAAAGC |
| pCV-NbPOLA2 -R | TCCTCGCCCTTGCTCACCATGTCGACTTATATACGAATAACTGAAGC |
| qNbPOLA2-F | TGGAGAAAGAAGTGAAGGGGAAG |
| qNbPOLA2-R | TGACAAATCACAGCTTCCGTG |
| qNbActin-F | CTTGAAACAGCAAAGACCAGC |
| qNbActin-R | CATCCTATCAGCAATGCCCG |
| qNbrRNA-F | GCGAGTAAACCCGTAAGG |
| qNbrRNA-R | GCTCAGGCATAGTTCACC |
